# A UAV-Based System for Validating a Backward Lagrangian Stochastic Model in a Dairy Cattle Farm

**DOI:** 10.3390/s25216733

**Published:** 2025-11-03

**Authors:** Alessio Mattia, Valentina Becciolini, Leonardo Conti, Marco Merlini, Patricia Ferreira Ponciano Ferraz, Gabriel Araújo e Silva Ferraz, Jacqueline Cardoso Ferreira, Franck Morais de Oliveira, Giuseppe Rossi

**Affiliations:** 1 Department of Agriculture, Food, Environment and Forestry, University of Florence, Via di San Bonaventura 13, 50145 Florence, Italy; alessio.mattia@unifi.it (A.M.); valentina.becciolini@unifi.it (V.B.); leonardo.conti@unifi.it (L.C.); marco.merlini@unifi.it (M.M.); 2 Department of Agricultural Engineering, Federal University of Lavras (UFLA), Lavras 37200-900, Brazil; patricia.ponciano@ufla.br (P.F.P.F.); gabriel.ferraz@ufla.br (G.A.e.S.F.); jacardosof@gmail.com (J.C.F.); franck.oliveira1@estudante.ufla.br (F.M.d.O.)

**Keywords:** drone, CO_2_ emissions, GHG, NDIR, compost barn, atmospheric dispersion, WindTrax

## Abstract

This study characterizes a compost-bedded pack barn of a dairy cattle farm in terms of CO_2_ emissions approximately 20 min after tilling under stable atmospheric conditions. Emission fluxes were calculated with the bLS model WindTrax, assessing modeled CO_2_ concentrations at two altitudes (5.0 m and 10.0 m ABGL) by comparing them with those measured by a UAV-based system at the same two altitudes. The UAV-based system was equipped with a low-cost self-engineered MSP (multi-sensor platform) containing an NDIR sensor for measuring concentrations and detecting environmental conditions, which were measured both by MSPs and commercial sensors. The input data were provided by the same sensors positioned on the ground (1.5 m ABGL), upwind and downwind with respect to the emission source. A sensitivity analysis of atmospheric stability in the bLS model yielded differences between median calculated emission fluxes for stable and unstable conditions from −0.020 to 0.034 g ∙ m^−2^ ∙ s^−1^. Mean percentage errors gave overestimates of 8–39% and 13–21% 5.0 m and 10.0 m ABGL. The RMSE also indicated overestimates ranging from 44 to 275 ppm. This is the first study to validate concentrations calculated by a bLS model at two altitudes by using a UAV-based system on a compost-bedded pack barn.

## 1. Introduction

The most recent published projections by the UN (United Nations) on global population growth show increases to 8.5 billion people for the year 2030 and to 9.7 billion people for the year 2050 [1]. Therefore, the agri-food industry should be efficient and sustainable in food and beverage production without harming the environment, people, or animals. In the year 2015, the UN approved Agenda 2030, where part of the SDGs (Sustainable Development Goals) is focused on improving air quality and taking action against climate change [2]. The WHO has estimated 6.7 million premature deaths per year caused by ambient outdoor and household air pollution [3]. In addition, pollutants affect climate change, since some of these are classified as GHGs (greenhouse gases), contributing to the greenhouse effect with different radiative impacts. At the European level, Directive 2008/50/EC aims to achieve cleaner air in Europe, which has been transposed to the national level, for example, by Italy with Legislative Decree 2010/155. Thus, in Italy, a pollutant-monitoring network managed by regional environmental protection agencies has been set up.

Agriculture is among the most impactful production sectors, and one of the most impactful agricultural activities in terms of atmospheric emissions is livestock farming. Global net anthropogenic GHG emissions for the year 2015 were estimated by [4] (p. 11) and reached a value of about 55 Gt CO_2_ eq. year^−1^. For the same year, the GLEAM (Global Livestock Environmental Assessment Model) estimated emissions from the livestock sector, which reached a value of 6.2 Gt CO_2_ eq. year^−1^, so livestock emissions represent 12% of global emissions. In livestock farming, cattle species contribute to sector emissions of 62%, i.e., 3.8 Gt CO_2_ eq. year^−1^. Within cattle farming, meat and milk production reach 40% (2.5 Gt CO_2_ eq. year^−1^) and 21% (1.3 Gt CO_2_ eq. year^−1^) of total sector emissions [5]. According to the same database, for total cattle farming emissions, CO_2_ emissions represent 26% (1.0 Gt CO_2_ eq. year^−1^). Furthermore, the other two major contributions to climate change from cattle farming are CH_4_ (methane) and N_2_O (nitrous oxide), at 61% (2.3 Gt CO_2_ eq. year^−1^) and 13% (0.5 Gt CO_2_ eq. year^−1^), respectively. Most of these emissions are attributed to enteric fermentation, manure management, and feed management. Another important source to assess is litter, because it is related to manure management and, consequently, hygiene and animal welfare.

UAVs (Unmanned Aerial Vehicles) are used for environmental monitoring, combining different solutions for selected types of analysis and sensor technologies. UAVs for environmental monitoring are used in several sectors with different applications: infrastructure [6,7,8], gas and oil [9,10,11], urban environments [12,13,14,15,16,17,18,19], wastewater treatment [20], research [21,22,23,24,25,26], landfills [27], industry [28], wildfires [29], vulcanology [30,31], and agriculture [32,33]. The common factor in these publications is the use of compact and lightweight sensors mounted on a UAV to comply with the maximum payload for the appropriate consumption and duration of UAV batteries. These sensors should measure gaseous and/or dusty concentrations and environmental conditions with the maximum atmospheric representativeness.

Most of the studies in the current literature use rotary-wing UAVs for environmental monitoring because of their high maneuverability, but the downwash problem has been highlighted, i.e., the downward turbulence generated by the rotors, which affects the sensor positioning on the aircraft used to maintain atmosphere representativeness. When determining where to place sensors above the UAV body through a computational fluid dynamics (CFD) analysis, ref. [7] (p. 21) stated that the optimal height above the drone body for sensor positioning is 90 cm, while [32] (p. 4) connected a long sampling tube to the concentration sensor to avoid air turbulence from the UAV blades, and [19] (p. 4) positioned the sampling inlet 60 cm above the drone rotors. On the other hand, ref. [23] (p. 5) placed sensors under the UAV body, but in this case, the measuring chamber had a downwash flow spoiler, reducing flow velocity and redirecting flow trajectory through concentration sensors. Sensors were positioned at the center of a UAV by [8] (p. 3) to mitigate the impact of propellers, based on the CFD analysis [34] of a quadrotor. Sensors were also placed under the UAV body by [25] (p. 483), but at the center of the UAV body itself. To justify the operational choices in the positioning of sensors in these studies, two CFD analyses have been reported, one referring to a hexacopter [35] and the other to a quadcopter [36]. Additionally, in a recent patent review [37], there is no explanation of a system or mechanism to deal with the downwash problem.

Moreover, ref. [38] (p. 4) stated that stationary monitoring with a measurement station on the ground provides data on emission variability at a single point, considering a low number of sources, limited to a specific place. Stationary monitoring alone cannot investigate the vertical variability in gaseous and dusty concentrations and environmental parameters. Instead, environmental monitoring with UAVs provides studies with a better spatio-temporal resolution, despite the analysis being limited by battery life, which is related to payload and the downwash problem. Many studies have focused on the characterization of the vertical profiles of atmospheric gaseous [8,16] or dusty [7,12,19,39] concentrations to understand transport mechanisms, complementing measurements of environmental conditions, while another work [17] determined the vertical profiles of both gaseous and dusty concentrations. Aerosol properties can vary significantly with altitude due to changes in temperature, humidity, atmospheric stability, solar radiation, and wind patterns [39] (p. 2); thus, gaseous and dusty concentrations are affected by meteorological conditions and turbulent mixing processes. For this reason, by studying the vertical distribution of pollutants, the accuracy of modeling atmospheric dispersion can be enhanced.

Previous studies flew UAV-based systems at several altitudes for obtaining vertical profiles of pollutants. The range of altitudes reached was from 15 m up to a maximum value of 2500 m ABGL (above ground level) [7,8,12,16,19,23,39]. The most widely detected pollutants included both gaseous species, such as VOCs, CO, CO_2_, CH_4_, NO_2_, O_3_, and SO_2_ [8,16,23], and particulate matter, including LDSA (lung-deposited surface area), ultrafine particles, PM_1_, PM_2.5_, PM_10_, and black carbon [7,12,19,39]. The sensors used to detect concentrations of gaseous pollutants included gas chromatographs, NDIR (non-dispersive infrared), and electrochemical methods, while particulate matter was measured using instruments such as multi-metric nanoparticle detectors, optical particle counters, diffusion size classifiers, portable microaethalometers, and condensation particle counters.

Regarding the methodologies used to analyze the detected concentrations, atmospheric dispersion models, particularly inverse dispersion modeling, are widely applied. Four CH_4_ leakage monitoring techniques were assessed in the USA by using a Gaussian plume model [9] (p. 4546). A Gaussian plume model was also employed to calculate CH_4_ leakage rates of undocumented orphan wells in the USA, using a fixed-wing UAV [11] (p. 19590). Particulate matter emission factors and emission fluxes from a feedlot and a dairy cattle farm in Texas, USA, were estimated with a model called Aermod [32] (p. 5). The Hysplit (Hybrid Single-Particle Lagrangian Integrated Trajectory) model was applied for performing trajectory directional analysis [16] (p. 5). WRF (weather research and forecasting) and CFD models were used in [14] (p. 2).

An NDIR sensor (Carbon3060S, Nanjing ZTweather Technology Co., Nanjing, China) was used in [20] (p. 4) and [23] to detect CO_2_. NDIR sensors are considered in a review by [38] (p. 2), along with other sensor types, such as amperometric, metal oxide chemiresistive, and photoionization detectors. These sensors showed high selectivity, sensitivity, and stability; fast response and recovery time; and low susceptibility to environmental variations. In the same review, four studies employing UAV-based systems equipped with NDIR sensors were reported [28,29,30,31]. Moreover, a strong correlation between UAV-based (NDIR) and ground-based (PiCam UV camera) CO_2_ measurements was found [31] (p. 743).

Among the techniques for gaseous analysis with UAV-based systems, this study selected an atmospheric dispersion model, i.e., WindTrax. Through an inverse modeling approach, WindTrax [40], a short-range bLS (backward Lagrangian Stochastic) model [41], generates many particles and follows them along their trajectories from sensors measuring gas concentrations back to the emission source for calculating emission fluxes. A graphical interface of the model is available in the associated software [42]. WindTrax is widely cited in the literature, especially in the agricultural sector. Most studies applying WindTrax v2.0 in agriculture focus on gas losses from various agricultural production sources, including fertilized soil management [43,44,45,46], anaerobic digestion [47,48], and livestock facilities, especially in relation to manure management [49,50,51,52].

CO_2_ was monitored in several studies [53,54], as it is classified as GHG. This paper could contribute to the development of a universal emission measurement protocol for updating “top-down” inventories and verifying “bottom-up” inventories [46]. The research gap identified in the current literature is the lack of assessment of the bLS model’s performance in predicting concentrations at the same altitude as UAV missions. Therefore, this study provides a direct comparison between predicted and observed concentrations obtained with a UAV-based system. Additionally, concentrations and environmental parameters measured by a UAV-based system could potentially be used in the WindTrax software tool with the Truck object [55] to enhance the accuracy of simulations that traditionally rely solely on input from stationary monitoring. However, this kind of assessment is beyond the objective of this paper. Overall, this paper focuses on assessing the bLS model in calculating predicted concentrations at the same altitude as a UAV mission.

Generally, a compost-bedded pack barn is designed and managed as a large free walk resting area under aerobic conditions with frequent mixing (1–3 times/day) performed using a tractor equipped with a disk harrow, a rotating tooth harrow, a cutter, or a subsoil plough. Frequent tilling incorporates fresh excrement, brings oxygen into the substrate, and promotes water evaporation. To achieve the latter, high ventilation rates are required to maintain fresh material, ensuring the thermal welfare of cows. The temperature within the litter increases with depth due to the composting process, producing NH_3_, CH_4_, N_2_O, and CO_2_, which are released into the atmosphere during tractor tilling [56]. A review assessing different manure management technologies, including anaerobic digestion, flushing, mechanical scrapers, and vacuum systems, reported that composting exhibits emission peaks immediately after tilling, since the gases trapped among solid particles in the litter are released into the atmosphere [57] (pp. 10–11). Over time, emissions tend to stabilize [58]; however, animal walking may compact the litter, reducing its porosity and consequently oxygen exchange. This promotes the formation of anaerobic sites, creating favorable conditions for methanogenic microorganisms.

The composting process has also been studied at laboratory scale with a static chamber, showing high GHG and NH_3_ emissions as temperature increases [59] (pp. 791–793). The advantages of using the compost-bedded pack barn include low-cost management, animal welfare, and high quality of milk production in dairy cattle farms. The highest NH_3_ and CH_4_ emissions were measured in Spain using gas chromatography before and during tilling in the summer season [60] (pp. 2–11). Additionally, it was reported that the rapid release of CO_2_ increases the pH of the substrate, tilting the balance of NH_3_ ↔ NH_4_^+^ toward N volatilization. This aspect affects the value of the compost-bedded pack barn material at the end of its life, which can be used as a fertilizer for cultivated fields.

CH_4_ and CO_2_ emissions were measured in 75 points of a resting area with a compost-bedded pack barn on a Brazilian dairy cattle farm, and spatial distribution maps were built using ordinary Kriging interpolation [61] (pp. 112–118). Animal thermal comfort was assessed using the THI, considering that mechanical ventilation can positively influence the composting process while also maintaining the THI below critical threshold values. Furthermore, CO_2_ is a dense gas that tends to accumulate in the lowest part of the resting area, which has important implications for animal welfare when cows are lying on litter, especially in closed livestock buildings. Regarding the current literature on compost-bedded pack barns, a research gap is identified: none of the publications have employed both a UAV-based system and an atmospheric dispersion model. In this type of analysis, the distance from the emission source, potential obstacles, and the flight altitude could be affected by the roof of the livestock building, where turbulence may compromise atmospheric representativeness, especially when considering the downwash problem.

The objective of this paper is to validate an atmospheric dispersion model based on a bLS approach using a UAV-based system, detecting CO_2_ concentrations with a self-engineered multi-sensor prototype. Two altitudes were investigated by mounting the MSP (multi-sensor platform) on a UAV at a Brazilian dairy cattle farm with a compost-bedded pack barn. Compared with stationary monitoring, this measurement protocol provides an affordable and autonomous emission measurement system to farmers that could potentially constitute a tool to obtain local top-down inventories, understanding emissions under different meteorological conditions throughout the year. UAV-based measurements can also complement stationary ground stations by detecting concentrations in the surrounding air.

In summary, the main research gaps addressed by this study are the assessment of bLS model performance in predicting concentrations at the same altitudes as a UAV mission, using a UAV-based system for environmental monitoring. This is the first study to validate concentrations predicted by a bLS model at two altitudes by using a UAV-based system for the environmental monitoring of CO_2_ emissions in the atmosphere from a compost-bedded pack barn in a dairy cattle farm.

## 2. Materials and Methods

### 2.1. Animals and Housing

**Figure 1:**
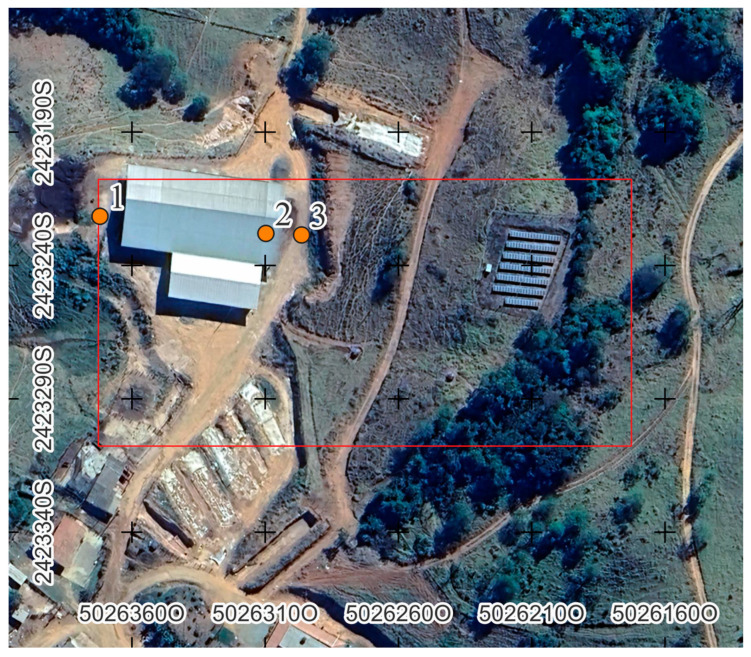
The dairy cattle farm where field trials were carried out, indicating the MSP positions (orange points); MSPs 1 and 2 were positioned on the ground, while MSP 3 was on the drone (WGS 84 reference system for longitude and latitude coordinates). The black marks (+) indicate distances of 50 m in latitude and longitude.

**Figure 2:**
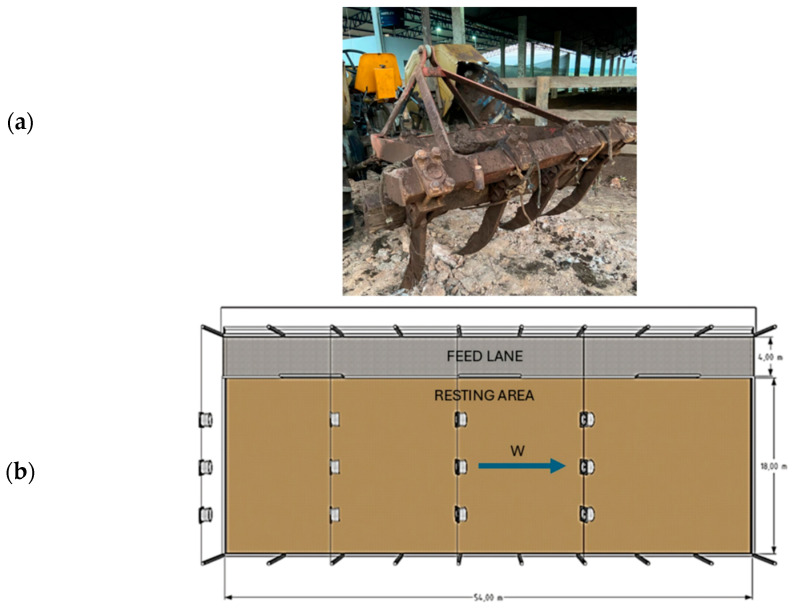
(**a**) The tooth harrow used for litter tilling; (**b**) functional area distribution inside the livestock building, where the forced ventilation direction is indicated by the blue arrow (from NW to SE).

The dairy cattle farm where field trials were conducted is located southeast of Belo Horizonte, approximately 30 km from Lavras in the federal state of Minas Gerais, Brazil. The farm comprises two buildings oriented from east to west (Figure 1). The smaller building contains the milking parlor and the milk room, while the larger one is the livestock building, which contains a compost-bedded pack in the resting area and a solid concrete floor in the feed lane. The building, designed for housing 80 cows, is open on all four sides except for the section occupied by the milking facility. The resting area spans 972.0 m^2^, providing 12.5 m^2^ available per cow. The bedding consists of a sawdust–manure mixture and has a depth of 65.0 cm. The solid grooved concrete floor in the feed lane is cleaned twice a day manually. Litter tillage is performed by a tractor equipped with a tooth harrow (Figure 2a) twice a day, in the morning at 06:00 a.m. and in the evening at 06:00 p.m., while cows are all moved in the feed lane. Twelve fans are installed 4.50 m above the litter to provide forced ventilation and remove moisture from the compost. Each fan has a diameter of 1.1 m, three propellers, a rotation speed of 950 revolutions per minute, and an airflow of 23,000 m^3^ ∙ h^−1^. The forced ventilation direction, from northeast to southwest, is illustrated in Figure 2b in relation to the internal distribution of functional areas of the livestock building.

**Figure 3:**
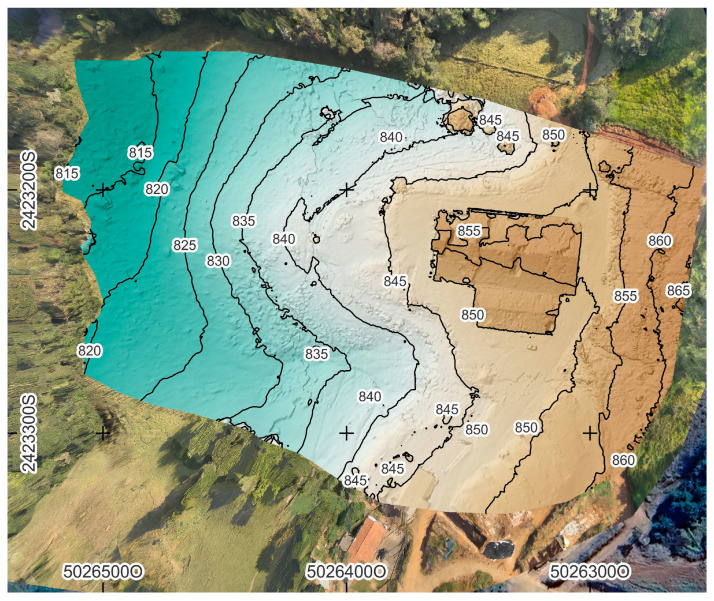
The DSM of the farm, including altitude contour lines above the ellipsoid level (WGS 84 reference system for longitude and latitude coordinates). The color scale represents the altitude from 812 to 870 m. The black marks (+) indicate distances of 100 m in latitude and longitude.

One of the assumptions of the WindTrax model is that the surface surrounding sources and sensors must be flat and free of obstructions [55] (p. 1). In this study, the soil around the livestock building had an average slope of approximately 20%. The slope was calculated from a DSM (Digital Surface Model) of the farm (Figure 3), obtained using the Zephyr v7.5 software tool [62] following UAV aero-photogrammetry operations. Altitude contours obtained with the QGIS v3.22.5 software tool [63] indicate the ellipsoidal height (WGS 84) in the DSM in Figure 3. Although WindTrax cannot implement the topographic surface of the soil, the present study focuses on assessing model performance through concentration sensors within MSPs placed close to the compost barn source, where the surface is nearly flat. Concentrations predicted by the bLS model at distances beyond the MSP locations are expected to exhibit larger error, as described in [64] (pp. 10–11).

Concerning the experimental conditions, it is stated that all the activities described in this paper were approved by the Ethics Committee on Animal Use of the Dean of research/UFLA on 26 October 2022, Protocol No. 044/22.

### 2.2. Multi-Sensor Platforms

Three low-cost MSPs, with a sampling frequency of five seconds, were used during an eight-day field trial on the dairy cattle farm. The trial characterized CO_2_ emissions from the compost-bedded pack barn by using the bLS model to assess its performance at two different heights above the ground: 5 and 10 m. Each MSP contains a CO_2_ concentration sensor (ppm) and sensors for measuring environmental parameters, including temperature (Celsius degree), pressure (Pa), and relative humidity (%). The CO_2_ concentration sensor (SCD30, Sensirion, Stäfa, Switzerland) integrated into the MSPs is an NDIR sensor. This type of sensor operates on the principle that certain gases absorb infrared light at specific wavelengths. CO_2_ exhibits a strong absorption peak in the mid-infrared region at approximately at 4.26 μm wavelength. Within the sensor, the infrared light passes through a measurement cell, where the amount of light absorbed by CO_2_ and the transmitted light are measured to calculate CO_2_ concentrations.

**Figure 4:**
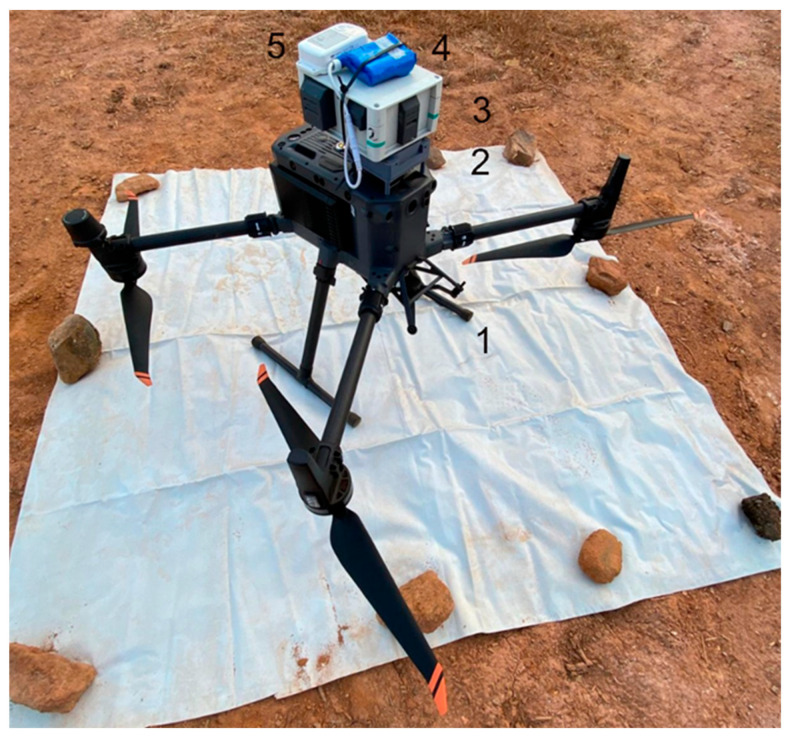
The UAV-based system and its parts: 1. the UAV; 2. the 3D-printed support; 3. the MSP; 4. the power bank; 5. the Temperature/RH Data Logger.

Temperature and relative humidity data are also provided by the CO_2_ sensor, thanks to an integrated module. The relatively low height of the module allows for easy integration for assembling sensors in an MSP, enabling the accurate and stable monitoring of CO_2_ in the air, temperature, and relative humidity. Furthermore, MSPs contain an absolute barometric pressure sensor (BMP280, Bosch Sensortec GmbH, Kusterdingen, Germany), which is compact and has low power consumption. Table 1 reports measuring range and accuracy for each detected parameter. The MSP solution is described in [65] (pp. 1051–1052), and the results of calibration tests in a controlled environment are provided in [66] (p. 931), particularly for the CO_2_ sensor (y = 67.517 + 0.855 ∙ x, R^2^ = 1, *p*-value < 0.001). While MSP weight is indicated as 1.20 kg, an upgraded version was used for field trials, maintaining the same CO_2_ sensor, removing some sensors and components, and reaching a weight of 0.70 kg. For the field trials, one MSP was mounted on a UAV (M350 RTK, DJI Sciences and Technologies Ltd., Beijing, China). This drone was selected for its large payload capacity, reaching 2.70 kg, sufficient to carry the sensors and ensure the stability of the UAV-based system. As noted in the literature, take-off weight is a critical parameter in UAV-based system environmental monitoring, as it influences battery life and, consequently, the sampling period duration. For this UAV-based system, a payload weight of 0.93 kg was reached, including a 3D-printed support to attach the MSP, a power bank to supply the MSP, and a Temperature/RH Data Logger (Hobo MX2301A, Onset Computer, Bourne, MA, USA), as shown in Figure 4. With this configuration, a maximum sampling period of 30 min per field trial was possible, keeping the UAV in the same position at two altitudes across several sampling days. As shown in Figure 4, the MSP was installed on top of the UAV body, with the measurement chamber inlet being 30 cm above the horizontal plane of propellers to minimize the effect of turbulence caused by downwash on concentration measurements.

Despite efforts for positioning the MSP on the UAV in one of the areas least affected by downwash, following solutions in the literature [7,8,19,23,25,32], it should be noted that UAV-based measurements cannot be considered entirely free from downwash effects. Therefore, caution is advised when interpreting the results reported in this paper, as the UAV-based system can be further improved. Field trials, including those reported here, can provide valuable insights for optimizing such measurements. In this context, data collected using the UAV-based system can serve as input for atmospheric dispersion models.

As shown in Table 1, an ultrasonic anemometer (Ultrasonic Portable Mini Wind Meter, Calypso Instruments EMEA, S.L, Coral Gables, Miami, FL, USA) was used for obtaining wind speed (m ∙ s^−1^) and wind direction (degrees from north) on the ground. The downwind sensors, including the other two MSPs and the anemometer, were positioned as close as possible to the litter source to ensure detectable wind speeds. During the experiment, natural wind speed in the area remained below 1 m ∙ s^−1^, which corresponds to the sensitivity threshold of the ultrasonic anemometer. To overcome this limitation, the sensors were placed near the building’s forced ventilation sources, which generated sufficient airflow for wind speed measurement. For all the sampling days, wind data from the ultrasonic anemometer were used for WindTrax simulations. The precise positions of the sensors were obtained from UAV-based aero-photogrammetry (Mavic 3 Enterprise, DJI Sciences and Technologies Ltd., Beijing, China), providing georeferenced photos in which sensors could be identified. Their geographical coordinates were then calculated using the DSM and imported into QGIS as background maps. For each field trial, the same sensor positions were consistently used.

### 2.3. Experimental Setup and Data Analysis

**Figure 5:**
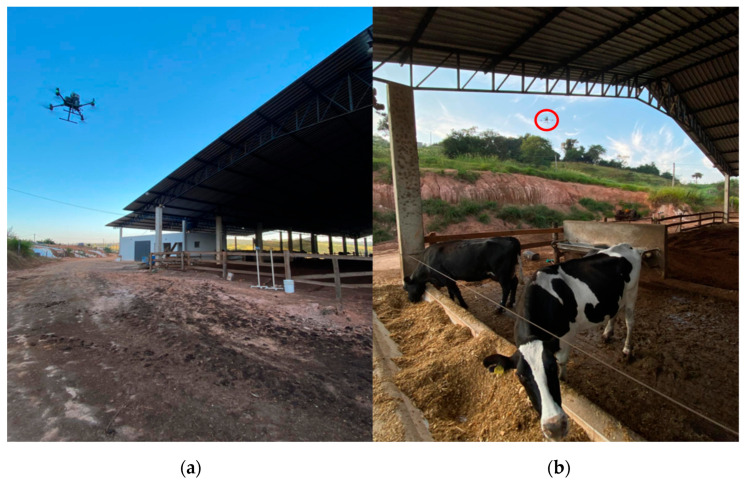
The operations performed by the UAV-based system for investigating the two altitudes in the dairy cattle farm: (**a**) 5.0 m ABGL; (**b**) 10.0 m ABGL. The red circle identifies the UAV-based system in flight.

In May 2024, the following experimental setup was applied at the dairy cattle farm: Eight field trials were conducted over eight days at approximately the same time each morning, from 05:40 to 06:40 a.m. Each trial included a 15 min sampling period after litter tilling. A 15 min averaging period is commonly recommended in the literature on WindTrax modeling and in the introductory manual [55]. The same three MSP positions were maintained during each field trial, as well as consistent sensor height for the MSPs and anemometers on the ground, i.e., 1.50 m from the ground level. Two MSPs, one upwind (MSP 1) and the other downwind (MSP 2), were used for obtaining input data for the WindTrax simulations. A third MSP (MSP 3) was mounted on a UAV that was flown downwind 5 m ABGL for the first four field trials and 10 m ABGL for the remaining field trials. The UAV-based system was used for comparing observed and modeled CO_2_ concentrations. As depicted in Figure 1, the ultrasonic anemometer was positioned on the ground close to the downwind MSP (MSP 2), while a hotwire anemometer was positioned close to the upwind MSP (MSP 1). Figure 5 shows the UAV-based system operating during regular farm activities.

**Figure 6:**
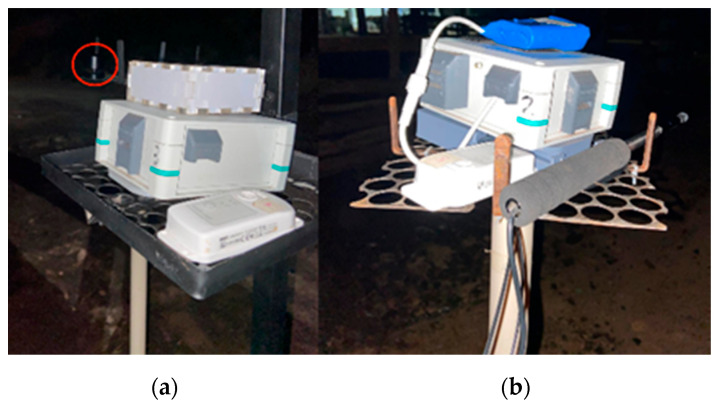
(**a**) The downwind measurement station on the ground, where the ultrasonic anemometer is indicated by the red circle; (**b**) the upwind measurement station on the ground, where the hotwire anemometer was positioned. A Temperature/RH Data Logger was positioned at both the stations on the ground.

In addition, for each field trial, Temperature/RH Data Loggers (Hobo MX2301A, Onset Computer, Bourne, MA, USA) were positioned near each MSP, including the one mounted on the UAV, comparing temperature and relative humidity data. Technical data of the Temperature/RH Data Logger sensors are shown in Table 1, while the sensors are shown in Figure 4 and Figure 6. Figure 6 depicts the sensors on the ground (1.5 m ABGL), positioned downwind and upwind, respectively. The hotwire anemometer shown in Figure 6b was used only for upwind measurements to assess wind conditions outside the influence of the forced ventilation system. For all the sampling days, the hotwire anemometer detected wind speed close to zero, indicating very weak winds outside the livestock building and strong atmospheric stability. These data were not used in the WindTrax simulations, as they were considered negligible. Furthermore, each MSP was equipped with designated air inlet and outlet directions, aligned with the forced ventilation direction during field trials. The UAV-based system was manually maintained in a hovering position downwind at the two fixed altitudes throughout the trials.

#### WindTrax Model Assumptions and Inputs

WindTrax is a short-range atmospheric dispersion model valid for averaging periods between 15 and 30 min, distances within approximately one kilometer, and flat and unobstructed terrain conditions. It solves a linear system of equations (Equations (1) and (2)):

a_11_ Q_1_ + a_12_ Q_2_ + … + a_1n_ Q_n_ + C_BG_ = C_1_ (1)

a_m1_ Q_1_ + a_m2_ Q_2_ + … + a_mn_ Q_n_ + C_BG_ = C_m_ (2)

C_BG_ is the background concentration, Q_j_ are the emission fluxes, C_i_ are the measured concentrations, and the coefficient a_ij_ relates Q_j_ and C_i_. If C_BG_ is unknown, an upwind concentration measurement is required, which was provided in this study via MSP 1. The model requires as inputs at least two concentration measurements; temperature, pressure, wind speed, and direction (via an anemometer); and the spatial coordinates of sources and sensors. It calculates the emission fluxes of the sources and then concentrations as outputs. The WindTrax software tool describes the microclimate thanks to the surface wind model based on Monin–Obukhov theory. For this reason, four parameters are required: the surface roughness length zo (related to the height of the elements covering the ground), the friction velocity u_*_ (the squared root of the covariance between the horizontal and vertical components of the wind), the Monin–Obukhov length L (atmospheric stability), and the mean horizontal wind direction θ [55,67]. The next section explains how these parameters were quantified, but a more extended description of the model is provided in the references [40,41], which also contain a study on sensor positioning according to the emission source [68].

The WindTrax software tool was used for calculating emission fluxes and concentrations from the compost-bedded pack barn. Other parameters required for simulations include the source area, surface roughness length, atmospheric stability, number of particles, and elevation of soil. The surface of the source was obtained by drawing in the software tool over geo-referenced images obtained in QGIS software. The selected surface roughness length z_o_ was 2.30 cm, i.e., short grass covering the surface around sources and sensors. Atmospheric stability was determined using Pasquill–Gifford classes. Under nighttime conditions, with wind speed being consistently of 2 ÷ 3 m ∙ s^−1^ and cloud cover being less than 50%, class F was assigned. The WindTrax software tool calculated the Monin–Obukhov length L from the selected Pasquill–Gifford class. The friction velocity u_*_ was also calculated by the model using the wind data. As noted in the introductory manual [55] (p. 1), increasing the number of particles reduces uncertainty; therefore, a high number of particles was selected (50,000) for the simulations. Soil elevation was set to zero, considering the pressure data provided by the MSPs.

Using data from MSPs 1 and 2, the T/RH Data Logger, and the Calypso anemometer, 50 CO_2_ emission fluxes per sampling day were calculated with the bLS model by using a fifteen-minute averaging period, resulting in a total of 400 emission fluxes. Descriptive statistics are provided for each field trial, including measured downwind concentrations and calculated emission fluxes. Additionally, a correlation matrix between input data and emission fluxes calculated by the bLS model is presented. Welch’s ANOVA test and then Dunn’s test with the Bonferroni *p*-value adjustment method were applied to compare the calculated emission fluxes for the same compost-bedded pack barn in three situations, i.e., before, during, and after litter tilling. It should be noted that emission fluxes before and during litter tilling were calculated in a previous study [69]. A sensitivity analysis is provided for modeled emission fluxes under stable (Pasquill–Gifford class F) and unstable (Pasquill–Gifford class B) atmospheric conditions. An additional 400 emission fluxes were calculated under unstable atmospheric conditions for comparison with those calculated under stable conditions. Given the stochastic feature of the bLS model, the several distributions of emission fluxes could assume normality or not, as verified with the Shapiro–Wilk test. Then, for each sampling day, the *t*-test and the Mann–Whitney U test were selected for comparing the calculated emission fluxes under stable and unstable conditions. Differences between medians are also provided.

Welch’s ANOVA test, followed by Dunn’s test with Bonferroni *p*-value adjustment, was applied for downwind concentrations detected at three altitudes: on the ground at 1.5 m and at 5.0 m and 10.0 m ABGL. For modeled concentrations, which were obtained using the mean calculated emission flux of each sampling day and the same input data used for calculating emission fluxes, concentration maps were generated over a 20,000 m^2^ surface 5.0 m ABGL for the first four sampling days and 10.0 m ABGL for the remaining days. The modeled concentrations at the two altitudes were compared with the Mann–Whitney U test. Furthermore, to assess model performance, measurements collected by the UAV-based system (MSP 3) at the same two altitudes were used to compute a mean ER (percentage error) between M (modeled) and O (observed) CO_2_ concentrations at the same position for each *i*th period:

ER (%) = (M_i_ − O_i_)/O_i_ (3)

Additionally, 95% confidence intervals at the heights from 0.5 to 12.0 m ABGL were calculated using the bLS model, and the mean values of the confidence intervals were used as modeled CO_2_ concentrations. For observed concentrations, the 15 min average values were used for the two sampling altitudes. 5.0 m ABGL for the first four days and 10.0 m ABGL for the remaining days. A quantitative validation of model performance is provided through the calculation of the RMSE (Root Mean Square Error):

(4) $$RMSE=\sqrt{\frac{1}{N}\sum_{i=1}^{n}{\left(M_{i}-O_{i}\right)}^{2}\;}$$

R [70] and Rstudio [71] were used for data analysis by selecting a set of library functions: packages like Openair [72,73], useful for air quality data analysis; Raster [74], for mapping and analyzing CO_2_ concentration data contained in raster files; Corrplot [75], for creating a correlation plot; and ggplot2 [76] for creating the following plots.

## 3. Results and Discussion

**Figure 7:**
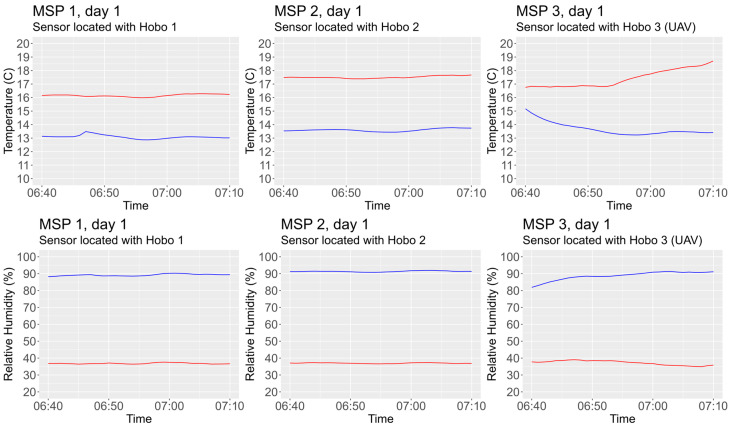
(**a**–**c**) Temperatures measured by MSPs (in red) and T/RH Data Loggers (in blue) on the first sampling day from 06:40 to 07:10 a.m.; (**d**–**f**) Relative humidity measured by MSPs (in red) and T/RH Data Loggers (in blue) on the first sampling day from 06:40 to 07:10 a.m.

**Scheme 1:**
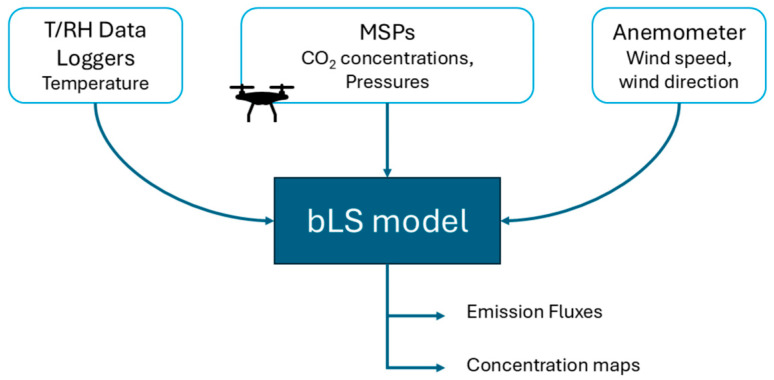
The variables measured by sensors used for simulations as input data for the bLS model.

By comparing temperature and relative humidity data obtained from the MSPs and T/RH Data Loggers, on the ground and in flight, the following observations were made: For all field trials, the Data Logger temperatures were lower, and the Data Logger relative humidities were higher than those measured by the MSPs, as can be seen in Figure 7 for the first sampling day. Graphs for the other sampling days are provided in the Supplementary Materials (Figures S1–S8). Two simple regression models were obtained, showing good agreement for temperature (y = −7.38 + 1.22 ∙ x, R^2^ = 0.86, *p*-value < 0.001) and poorer agreement for relative humidity (y = −892.19 + 50.11 ∙ x − 0.64 ∙ x^2^, R^2^ = 0.25, *p*-value < 0.001). Despite the good performance obtained by the temperature sensor inside the MSPs, to better represent the microclimate around the source, Data Logger temperatures and MSP pressures were selected for the simulations. The temperature detected by the Data Loggers was selected because of the positioning outside the MSP sampling chamber, providing observations that were more representative of atmospheric conditions. The pressure detected by the MSPs was selected because it was measured only by this sensor, despite significant differences in the relative humidity values recorded by the MSPs and the Data Loggers. All sensors and variables selected for each sensor for the bLS model are summarized in Scheme 1, which also shows the model outputs, discussed in detail later.

**Figure 8:**
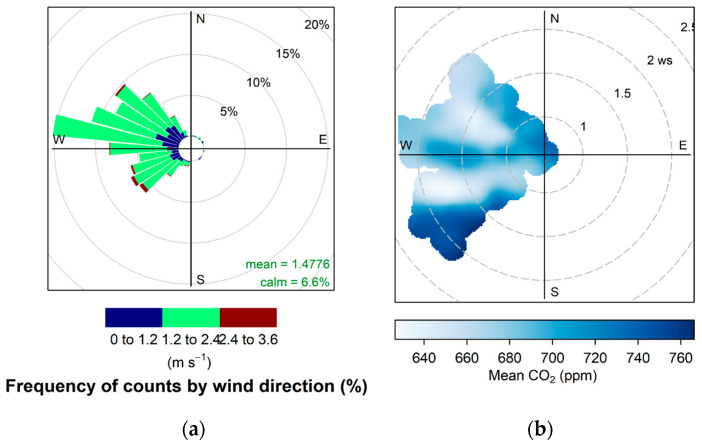
(**a**) The total wind rose for all sampling periods, using MSP 2 and the Calypso anemometer (wind speed classes (m ∙ s^−1^): 0–1.2 in blue, 1.2–2.4 in green, and 2.4–3.6 in dark red). (**b**) The polar plot (on the right) for all sampling periods, using MSP 2 and the Calypso anemometer (wind speed from 0 to 2.5 m ∙ s^−1^).

Table 2 presents the values of the variables measured by the MSPs and T/RH Dataloggers, averaged over 15 min periods to be used as input data for the bLS model. This table shows the time elapsed since litter tilling during the field trials. It is observed that upwind concentrations of CO_2_ were consistently lower than downwind concentrations for each sampling day, suggesting that other nearby emission sources did not contribute to the measurements. Although the ventilation system was always operational, the Calypso anemometer recorded non-uniform winds inside the livestock building during each sampling period, detecting winds with different directions and speeds (Figure 8), which affected pressure and temperature measurements. The maximum average wind speed occurred on the eighth sampling day, reaching a value of 1.83 m ∙ s^−1^, while the minimum, 1.14 m ∙ s^−1^, occurred on the second sampling day. Moreover, the wind rose and polar plot in Figure 8 indicate that higher CO_2_ concentrations at the MSP 2 position on the ground were measured when winds originated from southwest, with wind speeds exceeding 2.40 m ∙ s^−1^. However, these winds occurred with the lowest frequency (~20%) during each sampling day. Winds from west and northwest, which occurred most frequently (~80%), were associated with lower concentrations. This pattern suggests that the southwest area of the litter may contain a higher density of anaerobic microorganisms or more microbiological growth sites, although further microbiological monitoring of the litter would be required to confirm this.

### 3.1. CO_2_ Emission Fluxes

**Figure 9:**
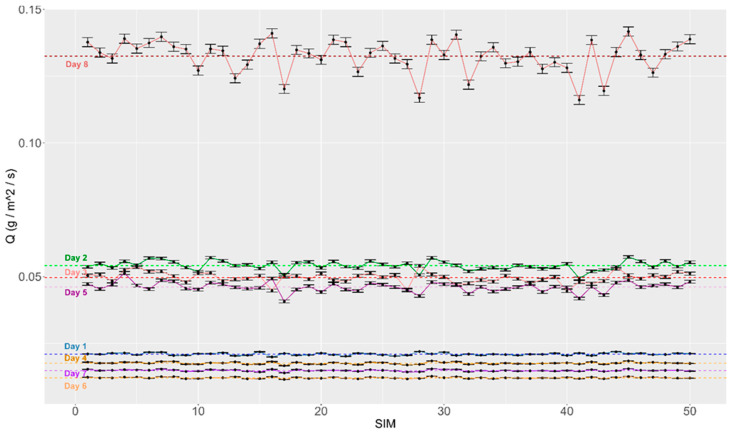
Scatter plot of all calculated CO_2_ emission fluxes (g ∙ m^−2^ ∙ s^−1^), including the mean values (dashed lines) and 95% confidence intervals (black error bars) for all the simulations performed for each sampling day.

The WindTrax software tool was used for calculating 50 CO_2_ emission fluxes after litter tilling, resulting in a total of 400 CO_2_ emission fluxes. A summary of the calculated values for each sampling day is provided in Table S1 of the Supplementary Materials, including minimum, first-quartile, median, mean, third-quartile, and maximum values. All the calculated CO_2_ emission fluxes can be observed in Figure 9 for all sampling days. The minimum CO_2_ emission flux occurred on the sixth day of measurements (0.01153 g ∙ m^−2^ ∙ s^−1^), while the maximum emission flux was calculated for the eighth sampling day (0.14170 g ∙ m^−2^ ∙ s^−1^), coinciding with the higher observed wind speeds. Across all sampling days, the overall average emission flux was 0.04351 ± 0.03730 g ∙ m^−2^ ∙ s^−1^, obtained approximately twenty minutes after litter tilling. Consequently, the corresponding standard deviation is relatively high.

CO_2_ emission fluxes were calculated by [58] (p. 61) for a compost-bedded pack barn with a moisture content of 49% in Kentucky twenty minutes after tillage; the measurements were performed using a static chamber, yielding a mean emission flux of 100.2 g ∙ m^−2^ ∙ h^−1^ (i.e., 0.02783 g ∙ m^−2^ ∙ s^−1^), a value comparable to those obtained in the present study despite differences in environment and calculation method. It should be noted that the moisture content was not measured in the present study. Mean CO_2_ emission fluxes before and during litter tilling were 0.03700 g ∙ m^−2^ ∙ s^−1^ and 0.06511 g ∙ m^−2^ ∙ s^−1^, respectively, based on four sampling days corresponding to the first four sampling days in the present study, so fully comparable due to the same environment and calculation methodology [69]. These data illustrate a trend in CO_2_ emission fluxes from compost-bedded pack barns: emissions peak during tilling and subsequently tend to stabilize and decrease twenty minutes after tilling, as animal activity compacts the litter [56,57,58]. The Shapiro–Wilk test confirmed that the distributions of emission fluxes calculated before, during, and after tilling did not follow normality (*p*-value < 0.001). Consequently, Welch’s ANOVA test was applied and then Dunn’s test with the Bonferroni *p*-value adjustment. The results confirmed significant differences between fluxes during and after tilling (*p*-value < 0.001) and between before and during tilling (*p*-value < 0.05), while no significant differences were found between after and before tilling.

**Figure 10:**
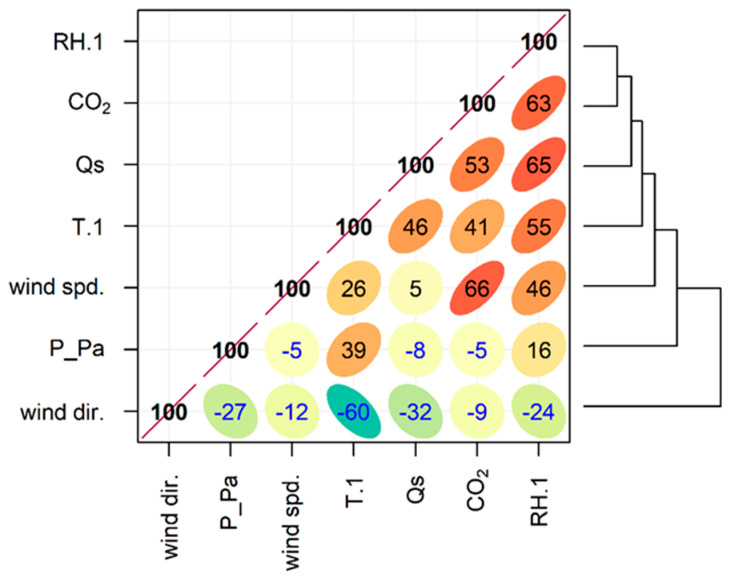
The dendrogram (black lines on the right side) and the correlation plot for the input data. The size of the circle and the intensity of the color in a cell correspond to the strength of the correlation (positives are coded from yellow to red and negatives from yellow to green); the absolute value of the correlation coefficient for two variables in pairs is black for positive correlations and blue for negative correlations. From the top, relative humidity (RH.1) detected by T/RH Data Loggers, CO_2_ concentration (CO_2_) detected by MSPs 1 and 2, emission fluxes (Qs) calculated by the bLS model, temperature (T.1) detected by T/RH Data Loggers, wind speed (ws) detected by the Calypso anemometer, pressure (P_Pa) detected by MSPs 1 and 2, and wind direction (wd) detected by the Calypso anemometer.

To investigate the relationships among the variables, a correlation matrix and a dendrogram are presented in Figure 10, showing Pearson correlation coefficients for the model input data considered in pairs. Data from stationary monitoring for each sampling day, i.e., those used to obtain input data for the model, were compiled into a dataset as follows: since sensors neither had the same sampling frequency nor started measuring at the same time, input data were averaged over a minute to join data frames from several sensors. As there were two sensors on the ground to obtain input data for the simulations, i.e., upwind and downwind, 15 observations per sensor on the ground were selected, obtaining a dataset of 30 observations per sampling day. In addition, since wind data were obtained only close to the downwind MSP (2), a median imputation was made to replace missing wind direction values, and a low-value (0.01 m ∙ s^−1^) imputation was made to replace missing values of wind speed for the upwind MSP (1). Finally, a random sampling of 30 emission fluxes out of the total 50 per sampling day was performed to create the response variable column, representing the calculated emission fluxes.

Starting from the environmental parameters used as input data for the emission flux calculation through the bLS model, the following results were obtained: The emission fluxes had a weak positive correlation (R = 0.46) with the temperatures detected by the T/RH Data Loggers, while they had a stronger positive correlation (R = 0.65) with the relative humidities detected by the same sensors. The temperature and relative humidity detected by the T/RH Data Loggers were also strongly positively correlated (R = 0.55), as in this case, water vapor was transported from the hotter litter toward the sensors by the turbulence generated by the ventilation system. Additionally, the emission fluxes also exhibited a strong positive correlation (R = 0.53) with the CO_2_ concentrations. The strongest positive correlation (R = 0.66) was observed between CO_2_ concentrations and wind speed, again reflecting the influence of the ventilation system.

Examining the relationship between emission fluxes and wind speed, a weak positive correlation (R = 0.05) was observed. This partially supports the initial hypothesis that the highest average wind speed could be responsible for the highest calculated emission fluxes for the eighth sampling day; however, it is insufficient to account for the calculation of other emission fluxes, as the relationship is not linear. For instance, the lowest mean emission flux (0.01213 g ∙ m^−2^ ∙ s^−1^) was calculated for sixth sampling day, which coincided with the second highest wind speed, i.e., 1.59 m ∙ s^−1^. Another factor to consider is the averaging period used in this study, i.e., fifteen minutes, which is recommended by the software author [67] to reduce the variability due to wind gusts in the atmosphere on short time scales. Other studies used this same averaging period [50], as well as shorter [77] or longer [78] intervals. Wind gusts should be considered in the emission flux calculation because, especially under unstable conditions, they could cause a reduction in the concentrations detected by downwind sensors, impacting calculated emission flux accuracy. Generally, WindTrax underestimates the emission rate under unstable conditions and overestimates it under stable conditions [50,54,79]. Some studies have filtered observations by removing low wind speeds, selecting data based on wind direction and atmospheric conditions characterized by high stability and instability [49,50,79].

Lower calculated emission fluxes have been reported as the atmospheric stability increase [80] (pp. 6–7). In the present study, stable atmospheric conditions (Pasquill–Gifford class F) were set in the software tool. However, wind gusts generated by the mechanical ventilation system in the livestock building tend to increase atmospheric instability, promoting CO_2_ dispersion and dilution processes. Indeed, as shown in Table 2, the maximum detected downwind concentration was not detected at the highest wind speeds (>1.50 m ∙ s^−1^). For example, on the second and fifth sampling days, the second- and fourth-highest emission fluxes were calculated, respectively.

The sensitivity analysis compared calculated emission fluxes under atmospheric stable and unstable conditions. After applying the Shapiro–Wilk test, the Mann–Whitney U test provided significant differences (*p*-value < 0.05) for the first, fourth, fifth, and eighth sampling days, while the *t*-test revealed significant differences (*p*-value < 0.05) for the second, third, sixth, and seventh sampling days. Table 3 presents the difference between medians of modeled emission fluxes under stable and unstable conditions, ranging from −0.020 to 0.034 g ∙ m^−2^ ∙ s^−1^.

### 3.2. CO_2_ Concentrations

**Figure 11:**
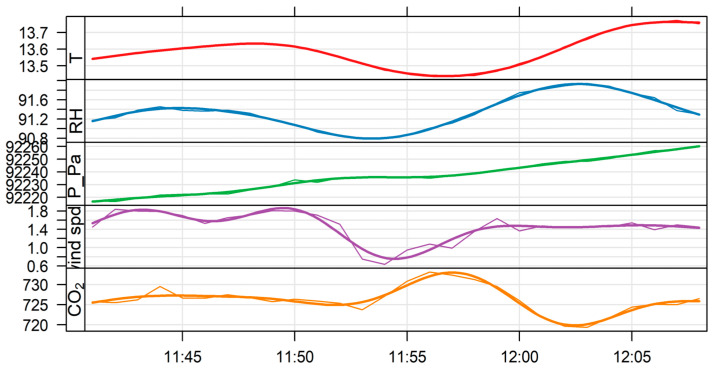
Detected environmental conditions (temperature (T) in Celsius degrees, relative humidity (RH) in %, wind speed (wind spd) in m ∙ s^−1^, and pressure (P_Pa) in Pascal) and CO_2_ concentrations (CO_2_) in ppm at the downwind position on the ground (1.5 m ABGL) on the first sampling day, showing smooth fit lines and a confidence interval of 98% obtained with the local regression method. The time format of the *x*-axis is CEST.

**Figure 12:**
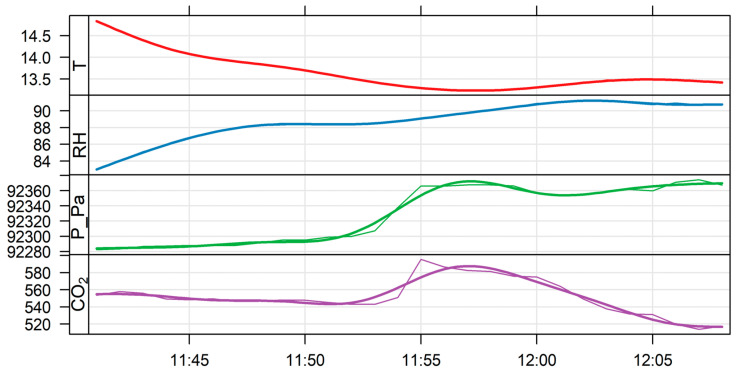
Detected environmental conditions (temperature (T) in Celsius degrees, relative humidity (RH) in %, and pressure (P_Pa) in Pascal) and CO_2_ concentrations (CO_2_) in ppm at the downwind position in flight (5.0 m ABGL) on the first sampling day, showing smooth fit lines and a confidence interval of 98% obtained with the local regression method. The time format of the *x*-axis is CEST.

Figure 11 shows the real-time environmental parameters (temperature, relative humidity, wind speed, and pressure) and CO_2_ concentrations detected at the downwind ground position on the first sampling day. Figure 12 shows the same parameters, except for wind speed detected in flight on the same day 5.0 m ABGL. It should be noted that for these graphs, the *x*-axis does not show the local time but follows the Italian time zone format (CEST). Since the sensors did not share the same sampling frequency or start time, input data were averaged over one-minute intervals to align datasets from several sensors. In both figures, the temperatures and relative humidities were measured by the T/RH Data Loggers, the CO_2_ concentrations and pressures by the MSPs, and the wind speeds by the Calypso anemometer, as these data were selected as input for the bLS simulations.

Observing CO_2_ concentrations at the two downwind positions (Figure 11 and Figure 12), higher values were measured at the ground level due to greater interception of the emission flux. This effect is attributed to the downward orientation of all 12 fans, which aim to reduce moisture in the compost-bedded pack barn. Furthermore, around 06:55 a.m., the wind speed dropped below 1 m ∙ s^−1^, increasing atmospheric stability. Consequently, concentration measured by MSP 2 (Figure 11) rose, reaching a peak of 735 ppm until the wind speed increased again, restoring conditions of atmospheric instability and reducing concentrations. This confirms a small short-term accumulation of CO_2_ in the building after tilling, due to its high molecular weight [61] and the occurrence of stable stratification. An increase in CO_2_ concentrations at the same time was also observed in flight 5.0 m ABGL (Figure 12) but with lower values due to dispersion and dilution at the higher altitude. These trends were consistent across all other sampling days (Supplementary Materials, Figures S9–S24).

Greater dispersion was observed 10.0 m ABGL, confirming the presence of a concentration gradient with altitude. Downwind concentrations measured at several altitudes (ground at 1.5 m, and 5.0 m and 10.0 m ABGL) during all the sampling days showed significant differences. The Shapiro–Wilk test confirmed that normality could not be assumed (*p*-value < 0.001) for all calculated concentrations at each altitude. Following the Shapiro–Wilk test, Welch’s ANOVA test was performed, followed by Dunn’s test with Bonferroni *p*-value adjustment. Significant differences were found between 5.0 m (median: 522.55 ppm) and 10.0 m ABGL (median: 490.22 ppm; *p*-value < 0.01). Even larger significant differences were observed (*p*-value < 0.001) between 1.5 m (median: 675.82 ppm) and 10.0 m ABGL, as well as between 1.5 m and 5.0 m ABGL. This analysis covered the period from the moment at which the UAV-based system reached its required position to the end of battery life and subsequent landing. Relative observation frequencies were 50% for ground measurements (211 observations), 25% for 5.0 m ABGL (103 observations), and 25% for 10.0 m ABGL (108 observations). These frequencies reflect that ground measurements were conducted on all sampling days, whereas flight measurements at the two selected altitudes were performed on half of the total sampling days.

**Figure 13:**
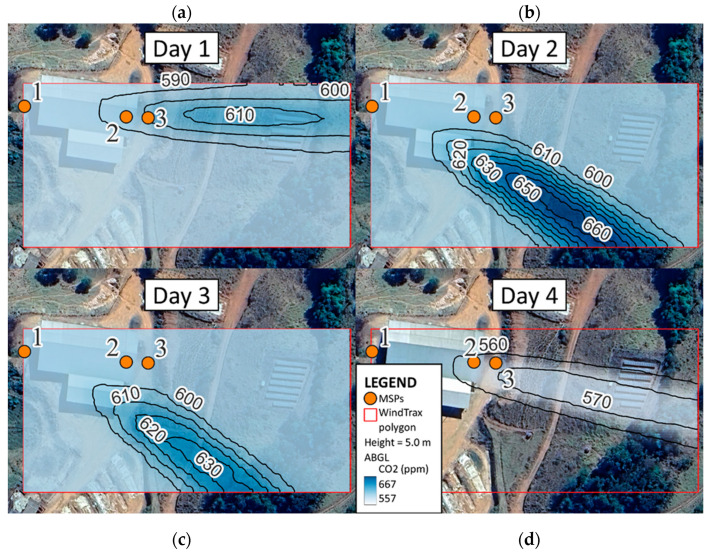
(**a**) CO_2_ atmospheric dispersions in ppm for the first sampling day at the height of 5.0 m ABGL; (**b**) CO_2_ atmospheric dispersions in ppm for the second sampling day at the height of 5.0 m ABGL; (**c**) CO_2_ atmospheric dispersions in ppm for the third sampling day at the height of 5.0 m ABGL; (**d**) CO_2_ atmospheric dispersions in ppm for the fourth sampling day at the height of 5.0 m ABGL. Orange dots represent the positions of the MSPs.

**Figure 14:**
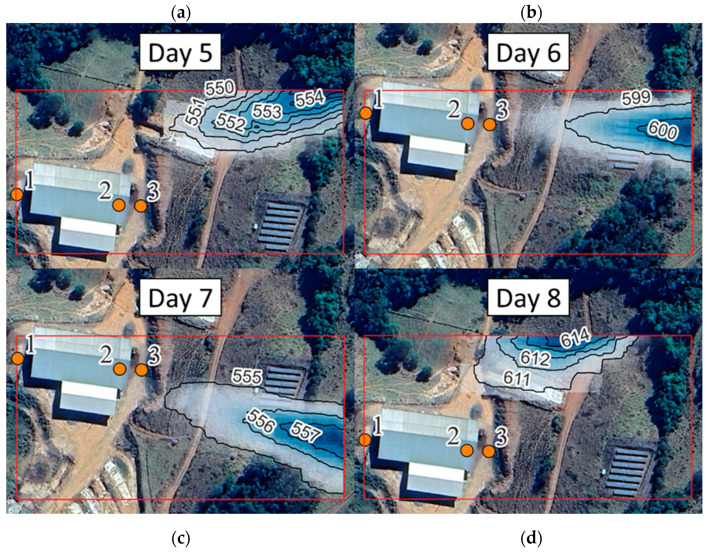
(**a**) CO_2_ atmospheric dispersions in ppm for the fifth sampling day at the height of 10.0 m ABGL; (**b**) CO_2_ atmospheric dispersions in ppm for the sixth sampling day at the height of 10.0 m ABGL; (**c**) CO_2_ atmospheric dispersions in ppm for the seventh sampling day at the height of 10.0 m ABGL; (**d**) CO_2_ atmospheric dispersions in ppm for the eighth sampling day at the height of 10.0 m ABGL. Orange dots represent the positions of the MSPs.

The modeled CO_2_ concentrations calculated using the bLS model are shown as concentration maps for 5.0 m ABGL in Figure 13 and for 10.0 m ABGL in Figure 14. The maps in Figure 13 all use the same scale to allow for a comparison of concentrations across the first four sampling days, while the maps in Figure 14 use different scales due to low variability. The CO_2_ atmospheric dispersion around the compost-bedded pack barn for each sampling day was represented as follows: Based on the wind direction (Figure 2b) a polygon of 20,000 m^2^ around the emission source was selected to capture the plume at both altitudes (Figure 1). Concentration maps were calculated within the polygon and exported by the WindTrax software tool as text files. These maps were then georeferenced using QGIS to generate the raster files shown in Figure 13 and Figure 14. Each raster file contains 7938 cells, with each cell representing a CO_2_ concentration calculated by the bLS model. It should be noted that the polygon was shifted northward for the fifth and eighth sampling days due to a measured wind from the southwest, which displaced the plume northward relative to the emission source.

The criticality of the model is its disregard for the topographic surface around the source and the sensors, meaning that the concentration maps do not represent the real CO_2_ atmospheric dispersion. In this study, the focus was on comparing the modeled concentrations with those measured by the UAV-based system (MSP 3), which was kept downwind at approximately the same position during each field trial, with altitude varying from 5.0 m to 10.0 m ABGL from the fifth to the eighth sampling day. Maximum values of modeled CO_2_ concentrations 5.0 m and 10.0 m ABGL are reported in Table S2. The analysis of the CO_2_ concentration maps shows that higher concentrations generally occurred 5.0 ABGL, reaching peaks of approximately 667 ppm and 635 ppm on the second and third sampling days, respectively. These peaks correspond to the second- and third-highest calculated emission fluxes. This result further confirms the altitude-based concentration gradient, highlighting dilution and dispersion processes at higher altitudes, as well as stable stratification of the target gas at lower altitudes, independently of the ventilation system. Additionally, higher modeled concentrations at 5.0 m ABGL were associated with winds from northwest, which occurred at higher frequency (Figure 8a).

At the height of 10.0 m ABGL, the two highest modeled concentrations, 616 ppm and 601 ppm, were reached on the eighth and sixth sampling days, respectively, corresponding in the simulations to the highest and eighth-highest calculated emission fluxes. This result was obtained under more unstable atmospheric conditions, with the two highest mean wind speeds measured 1.5 m ABGL, i.e., 1.83 m ∙ s^−1^ and 1.59 m ∙ s^−1^, producing comparable modeled concentrations between the two investigated altitudes. The explanation is that more unstable atmospheric conditions, characterized by more frequent wind gusts, promote upward transport of the target gas. Indeed, the highest modeled concentrations at 10.0 m ABGL were associated with winds from southwest, which occurred less frequently but with greater intensity (Figure 8a). The modeled concentrations at the two altitudes were compared using the Mann–Whitney U test after applying the Shapiro–Wilk test, yielding significant differences (*p*-value < 0.05).

**Figure 15:**
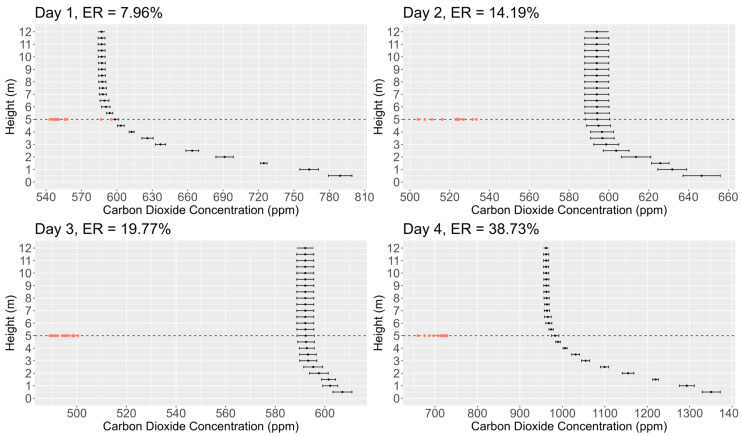
Vertical profile plots of modeled and observed CO_2_ concentrations for sampling days (**a**) 1, (**b**) 2, (**c**) 3, and (**d**) 4. Black intervals are 95% confidence intervals of modeled CO_2_ concentration, while red points are 1 min averages of observed CO_2_ concentrations at MSP 3. ER is the mean percentage error. The dashed green line represents the investigated altitude (5.0 m ABGL).

**Figure 16:**
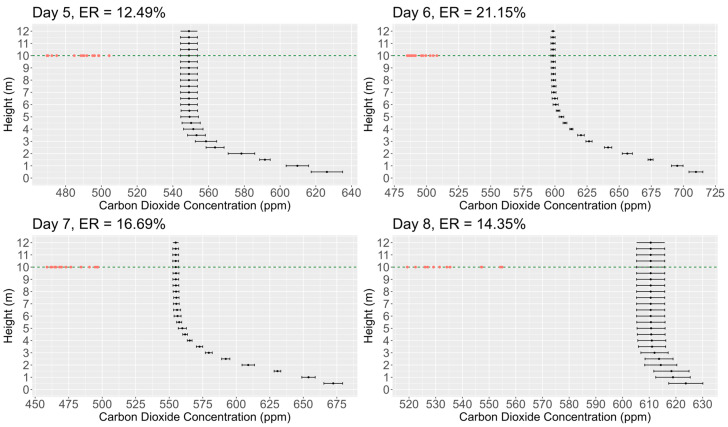
Vertical profile plots of modeled and observed CO_2_ concentrations for sampling days (**a**) 5, (**b**) 6, (**c**) 7, and (**d**) 8. Black intervals are 95% confidence intervals of modeled CO_2_ concentration, while red points are 1 min averages of observed CO_2_ concentrations at MSP 3. ER is the mean percentage error. The dashed green line represents the investigated altitude (10.0 m ABGL).

Using Equation 3, the mean ERs between modeled and observed CO_2_ concentrations at the same location, i.e., at the MSP 3 position (UAV-based system), were computed for each *i*th period. The bLS model calculated 95% confidence intervals at altitudes ranging from 0.5 to 12.0 m ABGL, and the mean values of these intervals were used as the modeled CO_2_ concentrations. For the observed concentrations, 15 min average values were used for the two sampling altitudes: 5.0 m ABGL for the first four days and 10.0 m ABGL for the remaining days. At the MSP 3 position, Figure 15 shows the predicted vertical profile plots for the first to the fourth sampling days, while Figure 16 shows the plots for the fifth to the eighth sampling days. In both Figures, the calculated 95% confidence intervals are indicated by black error bars for altitudes from 0.5 m ABGL to 12.0 ABGL, and fifteen mean observed CO_2_ concentrations (red points) were obtained by averaging measurements over 1 min intervals. Each vertical profile plot also indicates the mean ER, which ranged from approximately 8% to 39% 5.0 m ABGL and from 13% to 21% 10.0 m ABGL. Lower mean ER values 10.0 m ABGL were obtained due to the greater distance from the influence of the ventilation system to the UAV-based system, resulting in more stable atmospheric conditions, as set in the software tool.

During each field trial, the wind was measured downwind by a single ground-based sensor (1.5 m ABGL), showing more unstable atmospheric conditions due to wind gusts generated by the ventilation system inside the livestock building, compared with the atmospheric conditions set in the WindTrax software tool (Pasquill–Gifford class F, i.e., high stability). These frequent wind gusts affected all simulations over the limited 15 min averaging period because of rapid short-term changes in atmospheric stability near sunrise [55] (p. 2), overestimating the modeled concentrations at both investigated altitudes. Comparable results were found by other authors [50,54,79], although their studies focused on comparing emission fluxes calculated using different methodologies and sensors.

By applying Equation (4) and using the same data for the calculation of the mean ERs, RMSE was calculated for each sampling day (Table 4). Overestimated emission fluxes reported in the literature are directly linked to the overestimated concentrations obtained by the bLS model. Considering the environmental conditions during the field trials, RMSE values ranging from approximately 44 ppm to 275 ppm are notably high. These results suggest that more robust statistical validation could be achieved by improving the accuracy and representativeness of the input data used to calculate emission fluxes.

### 3.3. Limitations

A critical analysis of the limitation of applying the bLS model in a dairy cattle farm is provided. The results obtained should be contextualized with respect to the identified issues, including the model assumptions, operational limitations of the measurement system, and the capability to accurately describe the local atmospheric state. In this study, sensors were positioned close to the source, whereas in many previous studies, they were located further away. This choice was made for calculating emission rates in an area characterized by high density of obstacles and complex topographic conditions. Operational limitations related to the downwash issue of the UAV-based system must be considered when validating modeled concentrations. Implementing a new support structure that increases the distance between the rotor plane and the sensors could improve measurement accuracy.

Another key aspect concerns a better characterization of the local atmospheric state. Increasing the number of anemometers, both horizontally and vertically, would enhance the capability to accurately estimate atmospheric stability, currently approximated using Pasquill–Gifford classes. The implementation of ultrasonic anemometers capable of providing all three wind vector components should also be considered in the bLS model. While Eddy Covariance remains the most precise method for defining turbulence, it is expensive and requires multiple sensors [81]. Complementarily, placing anemometers on the UAV-based system could capture vertical wind profiles, further improving the model’s input data. To enhance model accuracy, it is also advisable to apply filters to the measured data, as recommended in recent studies [49,50,79], and to increase the number of particles in the simulations [50,79] despite the longer computation time, although 50,000 particles already provide reliable results.

This research study represents a first step toward establishing a methodology for quantifying atmospheric emissions from dairy cattle farms using the bLS model. This methodology could be extended to other processes in livestock farms, employing low-cost sensors and UAVs. By monitoring emissions seasonally and during regular farm activities, farmers could identify the most effective mitigation strategies or the best available techniques, evaluating their impact on the atmosphere and thus supporting an emission-based decision-making system.

## 4. Conclusions

This paper analyzed the performance of the bLS model WindTrax by calculating CO_2_ concentrations at two altitudes (5.0 m and 10.0 m ABGL), using stationary measurements (1.5 m ABGL) as input data, and comparing modeled concentrations with measurements obtained by a UAV-based system at the same altitudes. The agricultural process investigated was the management of a compost-bedded pack barn approximately 20 min after tilling. From the analysis, in terms of the average percentage error between modeled and measured concentrations, it is evident that a better quantification of on-site turbulence is necessary.

WindTrax is widely applied in agriculture and livestock farming, often combined with various technologies for measuring GHGs and environmental conditions. In this study, NDIR sensors were employed to measure CO_2_ concentrations. A critical factor for improving model accuracy is a more precise quantification of in situ atmospheric stability, which could be achieved by equipping the UAV-based system with an anemometer. In the present study, atmospheric stability was approximated using Pasquill–Gifford classes, with high stability (class F) being set in the software tool, because measurements were taken at night but close to sunrise and the average wind speed never exceeded 2.00 m ∙ s^−1^. The observed mean percentage errors between modeled and measured concentrations were largely due to increased atmospheric instability caused by wind gusts generated by the ventilation system in the short term. To mitigate the effect of wind gusts, longer averaging periods, 20 ÷ 30 min, would be beneficial. These findings are consistent with the correlations identified between emission fluxes and environmental conditions. Further studies across different times of the year are recommended to develop predictive models for seasonal emission patterns.

The UAV-based system, designed to place sensors in an area minimally affected by downwash, demonstrates potential for enhancing the accuracy of the atmospheric dispersion models. By enabling downwind measurements at multiple positions, it allows for better interception of the emission plume. However, before this innovative measurement system can be routinely applied, further validation is necessary to address the identified limitations and ensure reliable quantification of emissions.

## Figures and Tables

**Table 1:** Measuring range and accuracy of detected parameters by sensors inside MSPs and by the two types of anemometers (U—ultrasonic; HW—hotwire).

Parameter	Sensors	Measuring Range	Accuracy
Carbon dioxide	MSP	0 ÷ 5.000 ppm	±30 ppm
Temperature	MSP	−40 ÷ 70 °C	±(0.4 °C + 0.023 × (T [°C] − 25 °C))
Relative humidity	MSP	0 ÷ 100% RH	±3% RH
Pressure	MSP	300 ÷ 1.100 hPa	±1 hPa
Wind speed	U	1 ÷ 25 m ∙ s^−1^	±0.3 m ∙ s^−1^ at 10 m ∙ s^−1^
Wind direction	U	0 ÷ 359 °N	±1 °N
Temperature	Temperature/RH Data Logger	−40 ÷ 70 °C	±0.2 °C from 0 to 70 °C
Relative humidity	Temperature/RH Data Logger	0 ÷ 100% RH	±3% RH

**Table 2:** The 15 min average values of temperature (°C), pressure (Pa), CO_2_ concentration (ppm), wind speed (m ∙ s^−1^), and wind direction (degrees from north) measured upwind and downwind of the source by MSPs 1 and 2, the T/RH Data Logger, and the anemometer for each sampling day.

				MSP 1 (Upwind)			MSP 2 (Downwind)				
Day	Date (dd/mm/yy)	Time	Time After Tilling (min.)	Temp. (°C)	Press. (Pa)	CO_2_ (ppm)	Temp. (°C)	Press. (Pa)	CO_2_ (ppm)	WS (m ∙ s^−1^)	WD (° from N)
1	10 May 2024	06:56	23	13.14 ^H1^	92,257.79	586.21	13.57 ^H2^	92,228.27	727.13	1.49	270.95
2	13 May 2024	07:15	25	12.35 ^H3^	92,121.86	598.56	13.20 ^H1^	92,434.90	732.77	1.14	305.35
3	14 May 2024	07:15	21	10.59 ^H1^	92,150.12	595.48	11.48 ^H2^	92,131.36	665.19	1.65	309.29
4	15 May 2024	06:39	5	12.92 ^H3^	92,193.65	556.63	13.71 ^H2^	92,162.73	672.28	1.50	281.62
5	16 May 2024	07:15	15	14.66 ^H3^	92,943.47	547.54	15.68 ^H1^	92,219.53	654.41	1.38	245.89
6	17 May 2024	06:56	27	13.13 ^H3^	92,770.82	598.13	13.13 ^H2^	92,446.23	675.35	1.59	272.66
7	21 May 2024	07:15	14	13.12 ^H2^	92,513.28	555.58	14.13 ^H1^	92,485.73	650.01	1.36	286.02
8	28 May 2024	07:38	23	15.71 ^H1^	92,376.23	605.28	15.98 ^H2^	92,371.84	743.08	1.83	232.44

^H1, H2, H3^ are the temperatures measured by the T/RH Data Logger (Hobo MX2301A).

**Table 3:** Median values of emission fluxes (g ∙ m^−2^ ∙ s^−1^) under stable (class F) and unstable (class B) conditions for each sampling day, including the difference between them.

Day	Class F	Class B	Difference
1	0.021	0.041	−0.020
2	0.054	0.045	0.009
3	0.050	0.037	0.013
4	0.018	0.034	−0.016
5	0.046	0.042	0.004
6	0.012	0.024	−0.012
7	0.015	0.026	−0.011
8	0.134	0.100	0.034

**Table 4:** RMSE (ppm) calculated for each sampling day at two altitudes.

RMSE (ppm)							
Day 1	Day 2	Day 3	Day 4	Day 5	Day 6	Day 7	Day 8
46.62	74.42	97.83	275.12	61.92	104.74	80.40	77.44

## Data Availability

The raw data supporting the conclusions of this article will be made available by the authors upon request.

## Supplementary Materials

The following supporting information can be downloaded at: https://www.mdpi.com/article/10.3390/s25216733/s1, Figure S1: The temperatures and relative humidities measured by MSPs (in red) and T/RH Data Loggers (in blue) on the first sampling day, Figure S2: The temperatures and relative humidities measured by MSPs (in red) and T/RH Data Loggers (in blue) on the second sampling day, Figure S3: The temperatures and relative humidities measured by MSPs (in red) and T/RH Data Loggers (in blue) on the third sampling day, Figure S4: The temperatures and relative humidities measured by MSPs (in red) and T/RH Data Loggers (in blue) on the fourth sampling day, Figure S5: The temperatures and relative humidities measured by MSPs (in red) and T/RH Data Loggers (in blue) on the fifth sampling day, Figure S6: The temperatures and relative humidities measured by MSPs (in red) and T/RH Data Loggers (in blue) on the sixth sampling day, Figure S7: The temperatures and relative humidities measured by MSPs (in red) and T/RH Data Loggers (in blue) on the seventh sampling day, Figure S8: The temperatures and relative humidities measured by MSPs (in red) and T/RH Data Loggers (in blue) on the eighth sampling day, Table S1: Five-number summary (minimum, first-quartile, median, third-quartile, and maximum values), including the mean value of the fifty calculated CO_2_ emission fluxes (g ∙ m^−2^ ∙ s^−1^) for each sampling day, Figure S9: Detected environmental conditions (temperature in Celsius degrees, relative humidity in %, wind speed in m ∙ s^−1^, and pressure in Pascal) and CO_2_ concentrations in ppm at the downwind position on the ground on the first sampling day. The time format of the *x*-axis is CEST, Figure S10: Detected environmental conditions (temperature in Celsius degrees, relative humidity in %, and pressure in Pascal) and CO_2_ concentrations in ppm at the downwind position in flight (5.0 m ABGL) on the first sampling day. The time format of the *x*-axis is CEST, Figure S11: Detected environmental conditions (temperature in Celsius degrees, relative humidity in %, wind speed in m ∙ s^−1^, and pressure in Pascal) and CO_2_ concentrations in ppm at the downwind position on the ground on the second sampling day. The time format of the *x*-axis is CEST, Figure S12: Detected environmental conditions (temperature in Celsius degrees, relative humidity in %, and pressure in Pascal) and CO_2_ concentrations in ppm at the downwind position in flight (5.0 m ABGL) on the second sampling day. The time format of the *x*-axis is CEST; Figure S13: Detected environmental conditions (temperature in Celsius degrees, relative humidity in %, wind speed in m ∙ s^−1^, and pressure in Pascal) and CO_2_ concentrations in ppm at the downwind position on the ground on the third sampling day. The time format of the *x*-axis is CEST, Figure S14: Detected environmental conditions (temperature in Celsius degrees, relative humidity in %, and pressure in Pascal) and CO_2_ concentrations in ppm at the downwind position in flight (5.0 m ABGL) on the third day. The time format of the *x*-axis is CEST, Figure S15: Detected environmental conditions (temperature in Celsius degrees, relative humidity in %, wind speed in m ∙ s^−1^, and pressure in Pascal) and CO_2_ concentrations in ppm at the downwind position on the ground on the fourth sampling day. The time format of the *x*-axis is CEST, Figure S16: Detected environmental conditions (temperature in Celsius degrees, relative humidity in %, and pressure in Pascal) and CO_2_ concentrations in ppm at the downwind position in flight (5.0 m ABGL) on the fourth day. The time format of the *x*-axis is CEST, Figure S17: Detected environmental conditions (temperature in Celsius degrees, relative humidity in %, wind speed in m ∙ s^−1^, and pressure in Pascal) and CO_2_ concentrations in ppm at the downwind position on the ground on the fifth sampling day. The time format of the *x*-axis is CEST, Figure S18: Detected environmental conditions (temperature in Celsius degrees, relative humidity in %, and pressure in Pascal) and CO_2_ concentrations in ppm at the downwind position in flight (10.0 m ABGL) on the fifth day. The time format of the *x*-axis is CEST, Figure S19: Detected environmental conditions (temperature in Celsius degrees, relative humidity in %, wind speed in m ∙ s^−1^, and pressure in Pascal) and CO_2_ concentrations in ppm at the downwind position on the ground on the sixth sampling day. The time format of the *x*-axis is CEST, Figure S20: Detected environmental conditions (temperature in Celsius degrees, relative humidity in %, and pressure in Pascal) and CO_2_ concentrations in ppm at the downwind position in flight (10.0 m ABGL) on the sixth day. The time format of the *x*-axis is CEST, Figure S21: Detected environmental conditions (temperature in Celsius degrees, relative humidity in %, wind speed in m ∙ s^−1^, and pressure in Pascal) and CO_2_ concentrations in ppm at the downwind position on the ground on the seventh sampling day. The time format of the *x*-axis is CEST, Figure S22: Detected environmental conditions (temperature in Celsius degrees, relative humidity in %, and pressure in Pascal) and CO_2_ concentrations in ppm at the downwind position in flight (10.0 m ABGL) on the seventh day. The time format of the *x*-axis is CEST, Figure S23: Detected environmental conditions (temperature in Celsius degrees, relative humidity in %, wind speed in m ∙ s^−1^, and pressure in Pascal) and CO_2_ concentrations in ppm at the downwind position on the ground on the eighth sampling day. The time format of the *x*-axis is CEST, Figure S24: Detected environmental conditions (temperature in Celsius degrees, relative humidity in %, and pressure in Pascal) and CO_2_ concentrations in ppm at the downwind position in flight (10.0 m ABGL) on the eighth day. The time format of the *x*-axis is CEST, Table S2: Five-number summary (minimum, first-quartile, median, third-quartile, and maximum values), including the mean value of the fifty calculated CO_2_ concentrations (ppm) for each sampling day.
