# Supplementary material for: A UAV-Based System for Validating a Backward Lagrangian Stochastic Model in a Dairy Cattle Farm
Source: Sensors (Basel). 2025 Nov 3;25(21):6733. doi: 10.3390/s25216733 (PMC12610183; doi:10.3390/s25216733)
Supplement: Supplementary file 1 [file sensors-25-06733-s001.zip › sensors-3897683-supplementary.pdf]

Supplementary Material

Detected temperatures and Relative Humidities for each sampling day with MSPs and Temperature/RH Data Loggers

Day 1

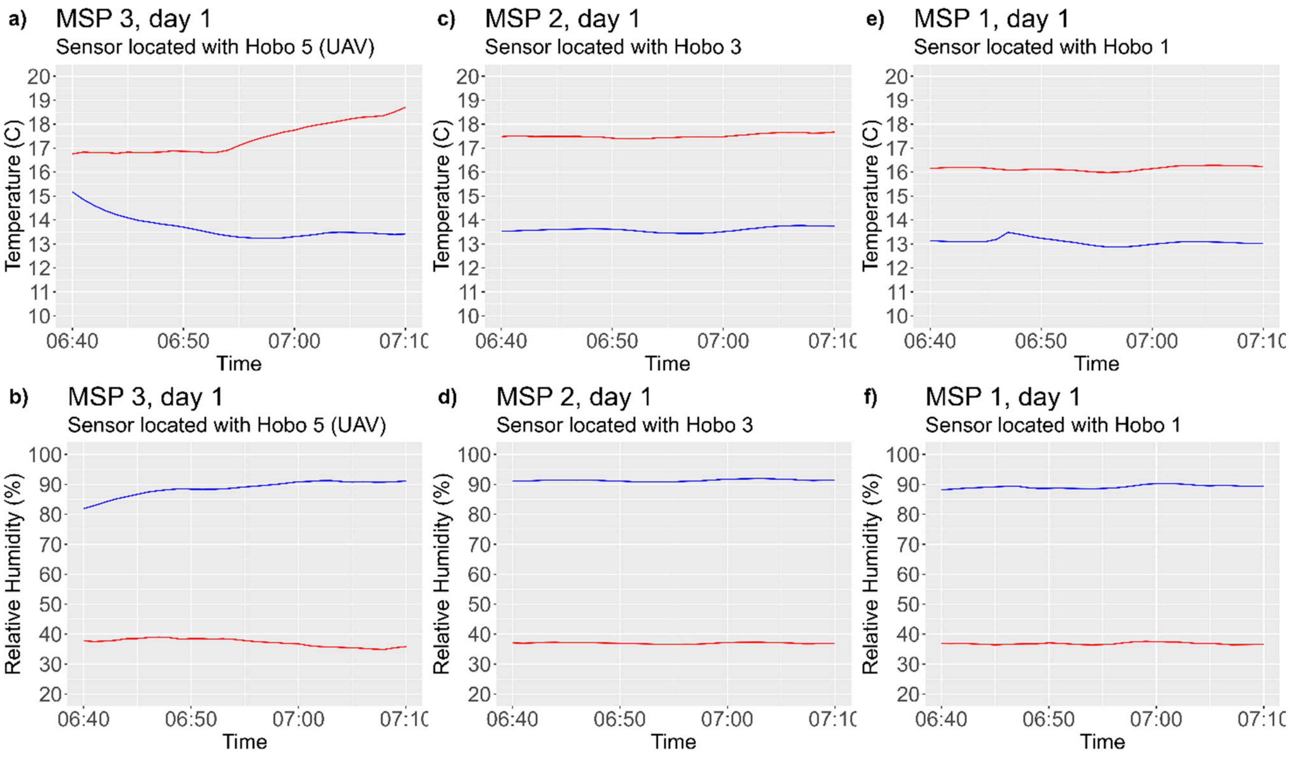

Figure S1. Measured temperatures and relative humidities by MSPs (in red) and T/RH Data Loggers (in blue) on the first sampling day.

Day 2

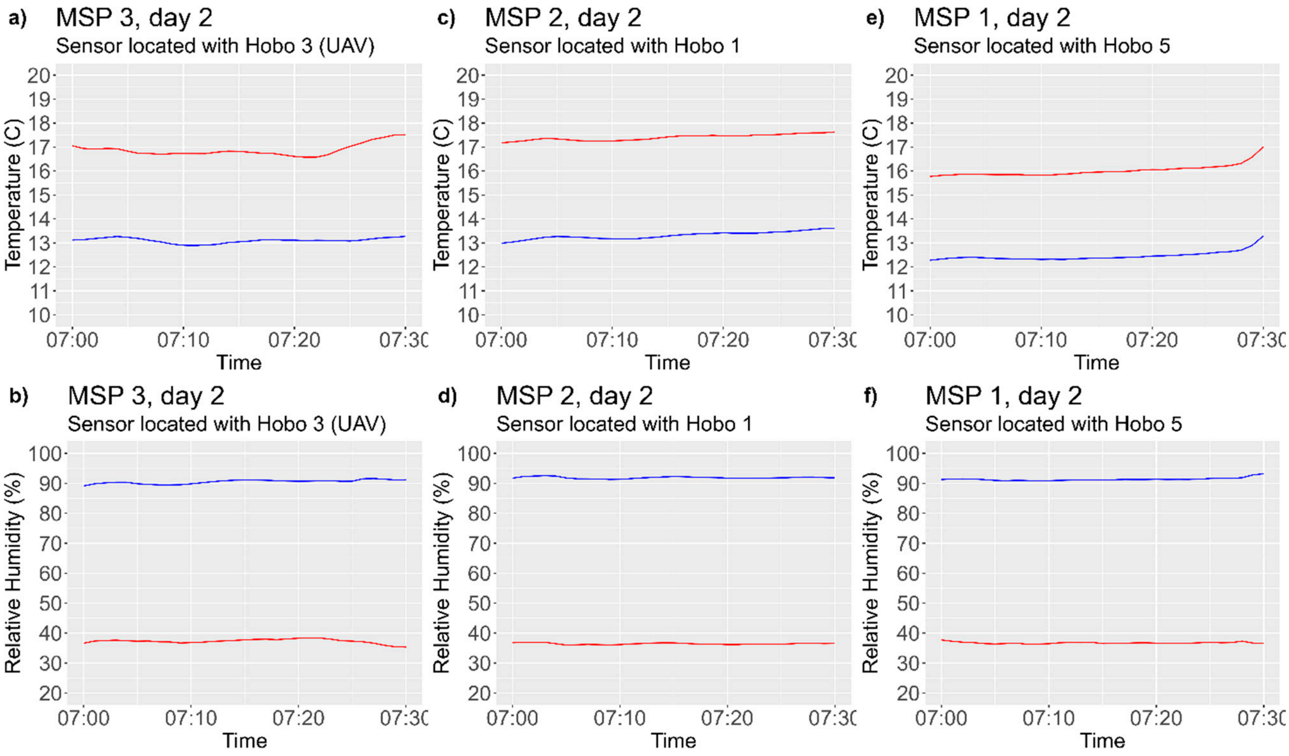

Figure S2. Measured temperatures and relative humidities by MSPs (in red) and T/RH Data Loggers (in blue) on the second sampling day.

Day 3

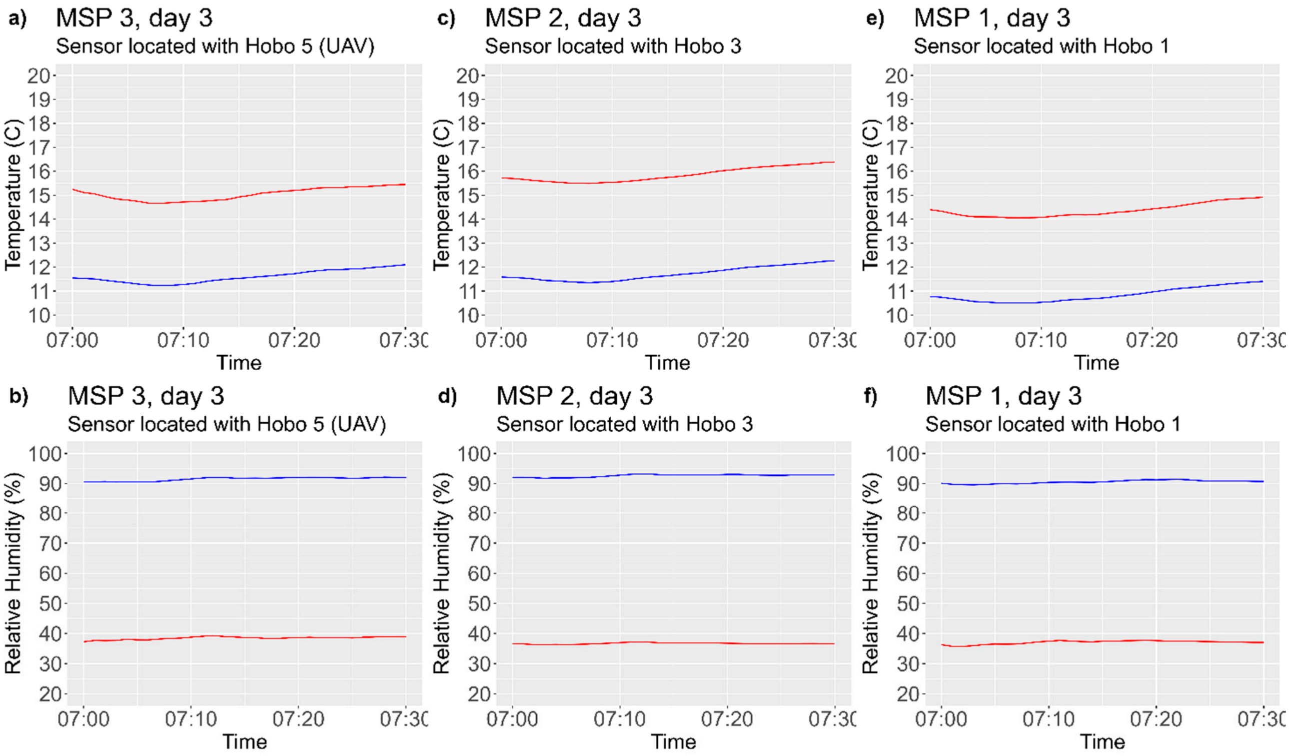

Figure S3. Measured temperatures and relative humidities by MSPs (in red) and T/RH Data Loggers (in blue) on the third sampling day.

Day 4

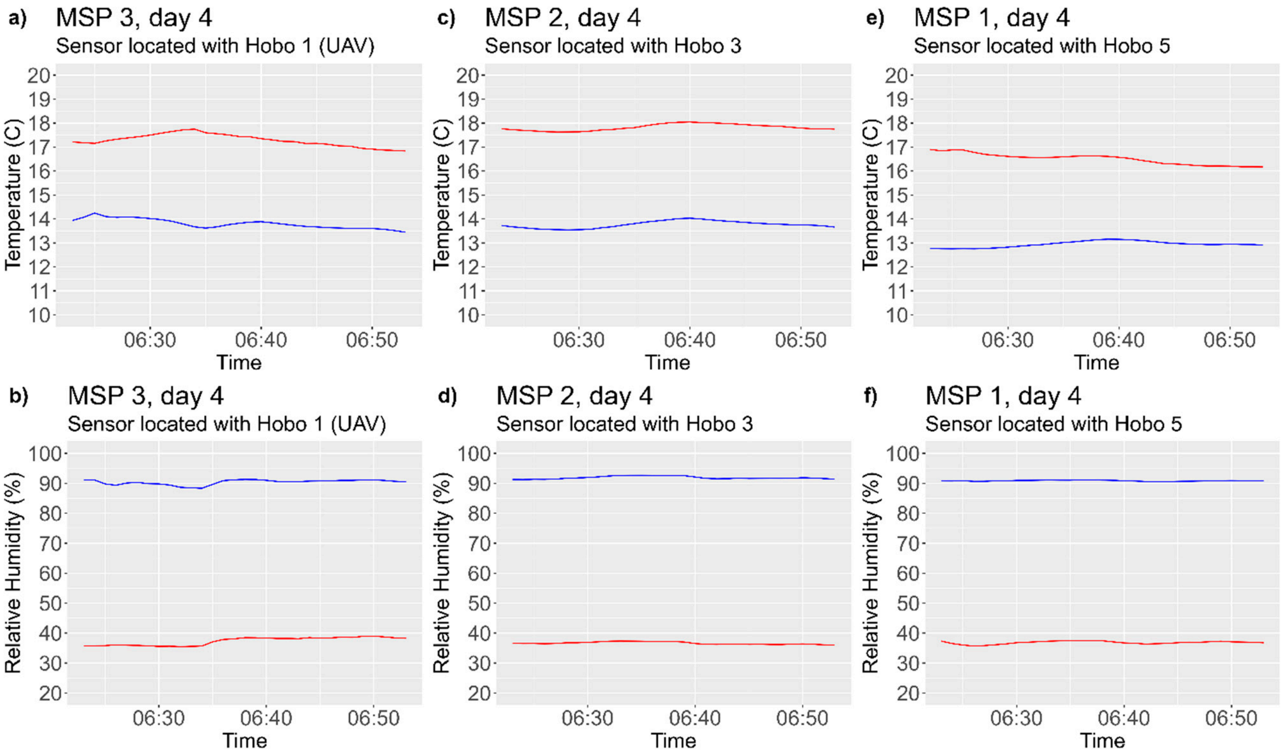

Figure S4. Measured temperatures and relative humidities by MSPs (in red) and T/RH Data Loggers (in blue) on the fourth sampling day.

Day 5

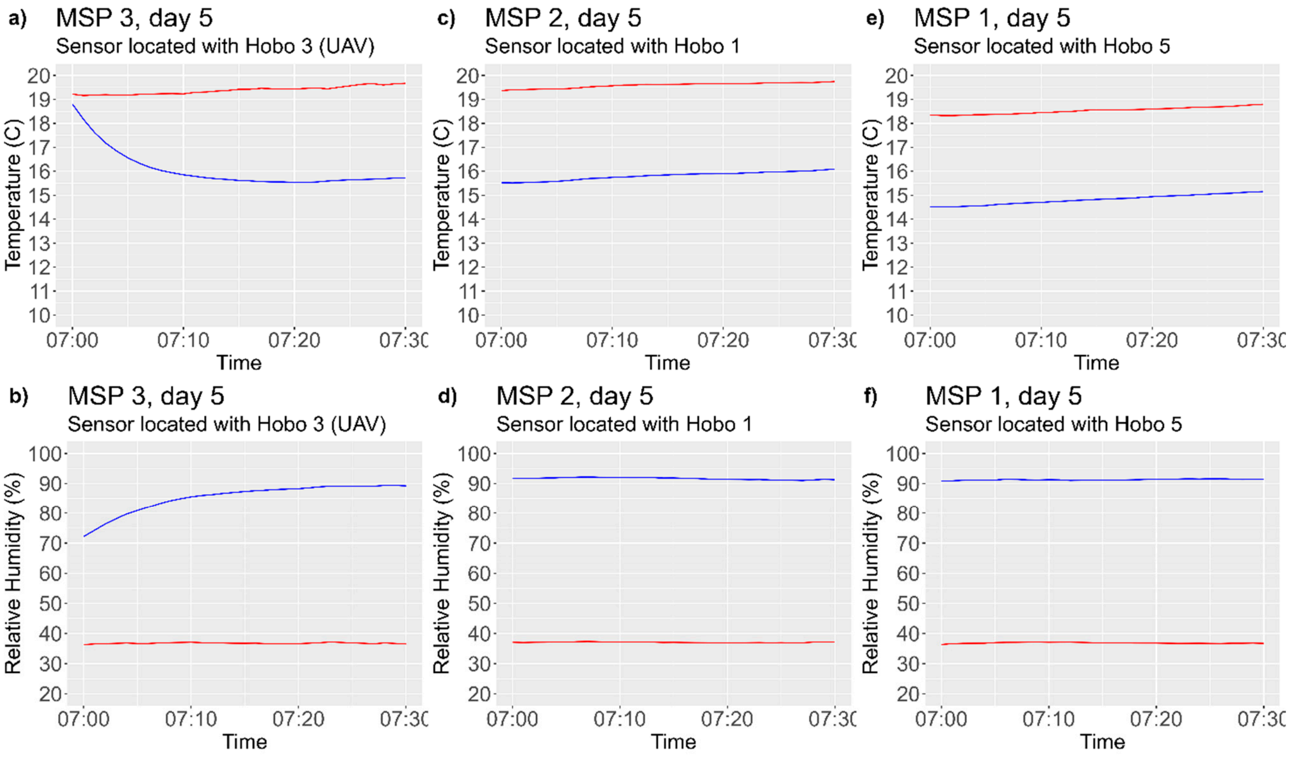

Figure S5. Measured temperatures and relative humidities by MSPs (in red) and T/RH Data Loggers (in blue) on the fifth sampling day.

Day 6

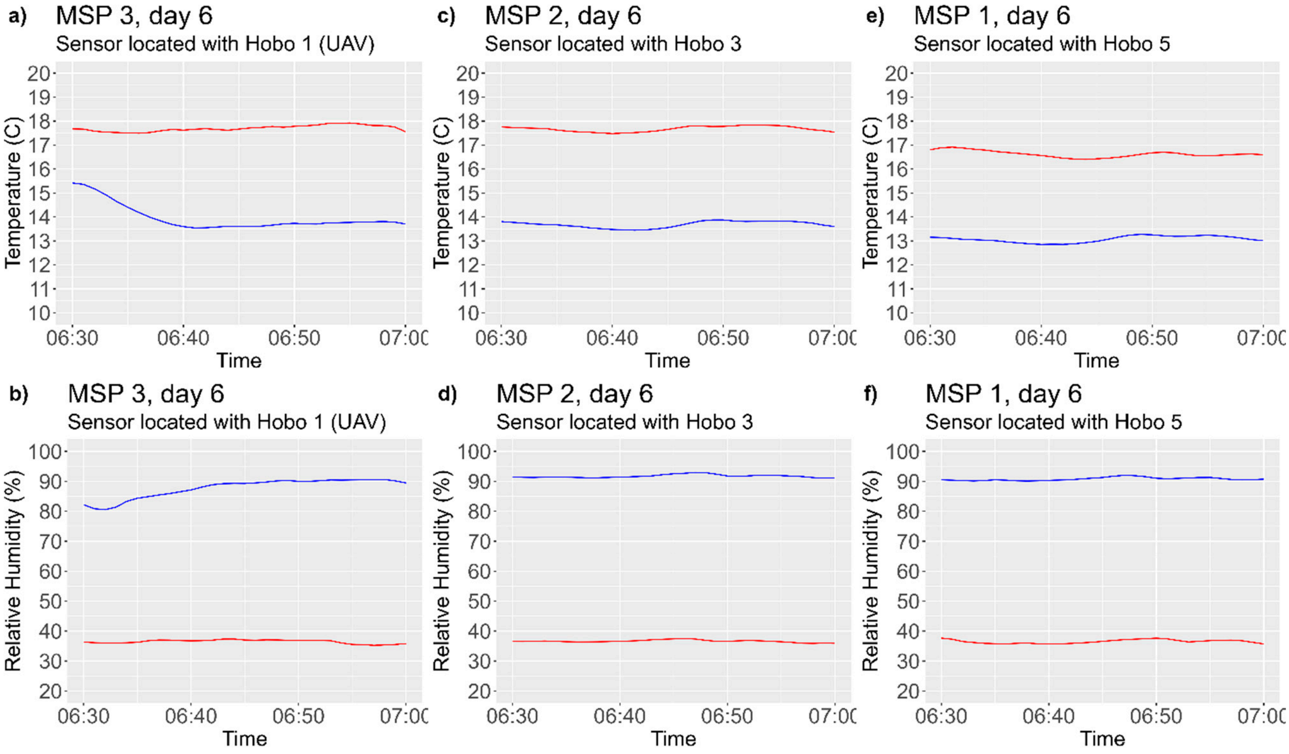

Figure S6. Measured temperatures and relative humidities by MSPs (in red) and T/RH Data Loggers (in blue) on the sixth sampling day.

Day 7

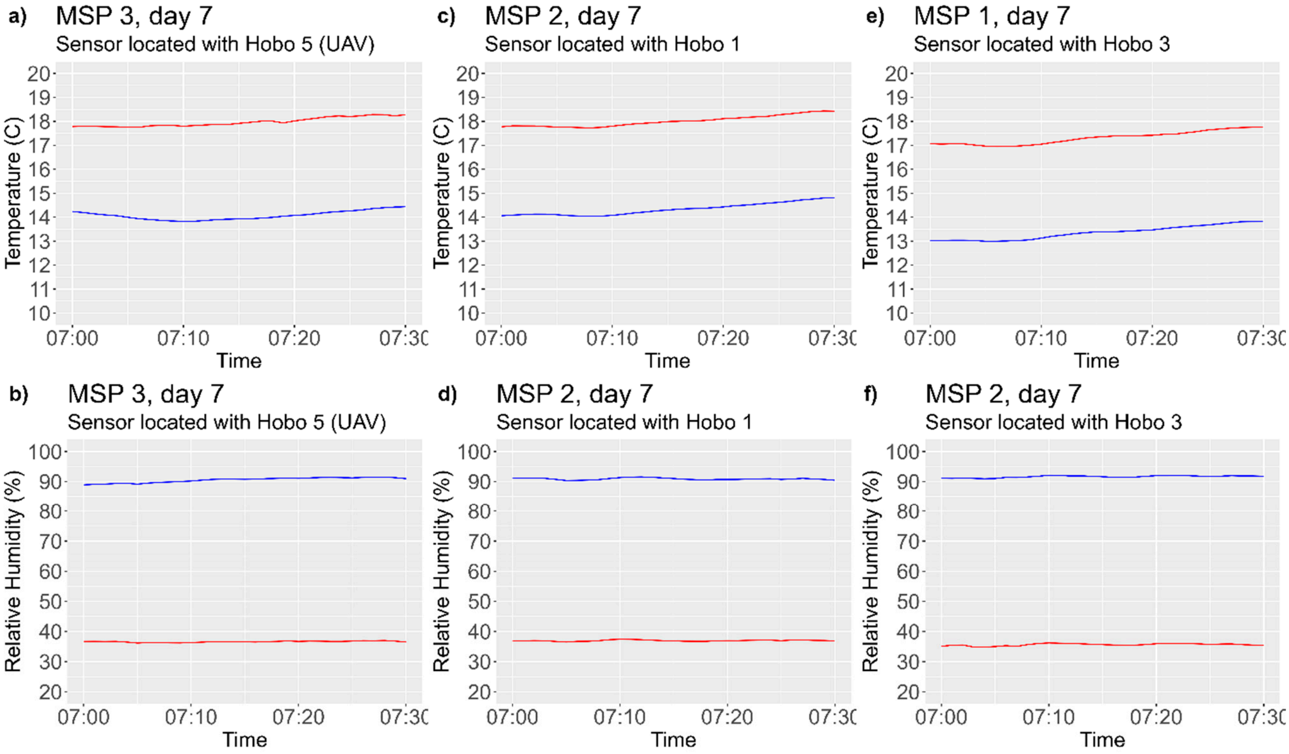

Figure S7. Measured temperatures and relative humidities by MSPs (in red) and T/RH Data Loggers (in blue) on the seventh sampling day.

Day 8

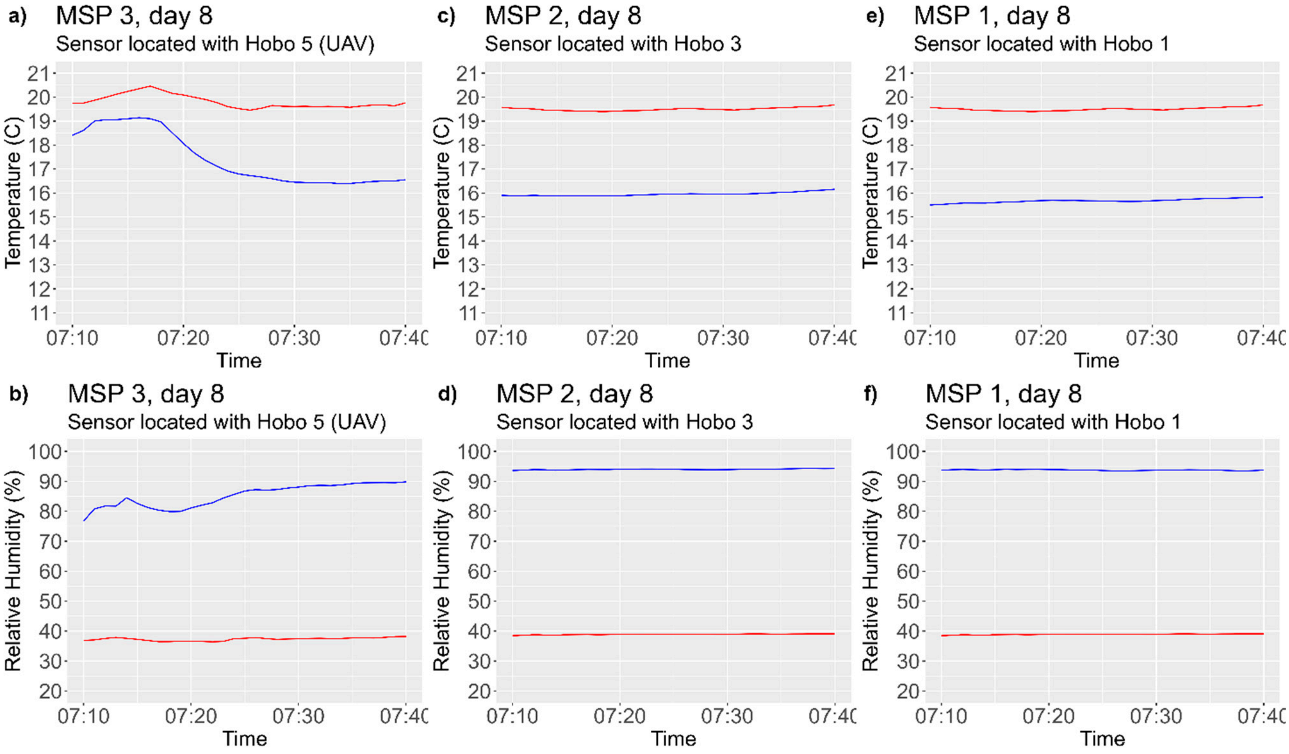

Figure S8. Measured temperatures and relative humidities by MSPs (in red) and T/RH Data Loggers (in blue) on the eighth sampling day.

Calculated CO_2 emission fluxes for each sampling day

Table S1. The five-number summary (minimum, first quartile, median, third quartile, and maximum values), including the mean value of the fifty calculated CO_2 emission fluxes (g m^{-2} s^{-1}) for each sampling day.

Statistics	Day							
	1	2	3	4	5	6	7	8
Minimum	0.01994	0.04935	0.04461	0.01666	0.04071	0.01153	0.01398	0.11610
1st quartile	0.02060	0.05319	0.04831	0.01727	0.04522	0.01191	0.01464	0.12960
Median	0.02100	0.05414	0.04989	0.01764	0.04614	0.01212	0.01486	0.13380
Mean	0.02100	0.05414	0.04966	0.01763	0.04614	0.01213	0.01487	0.13250
3rd quartile	0.02134	0.05545	0.05078	0.01792	0.04722	0.01233	0.01511	0.13690
Maximum	0.02198	0.05734	0.05404	0.01845	0.05129	0.01270	0.01552	0.14170

Detected environmental conditions (temperature, relative humidity, wind speed, and pressure), and CO_2 concentrations at the downwind positions: on the ground and in flight (5.0 m and 10.0 m ABGL).

Day 1

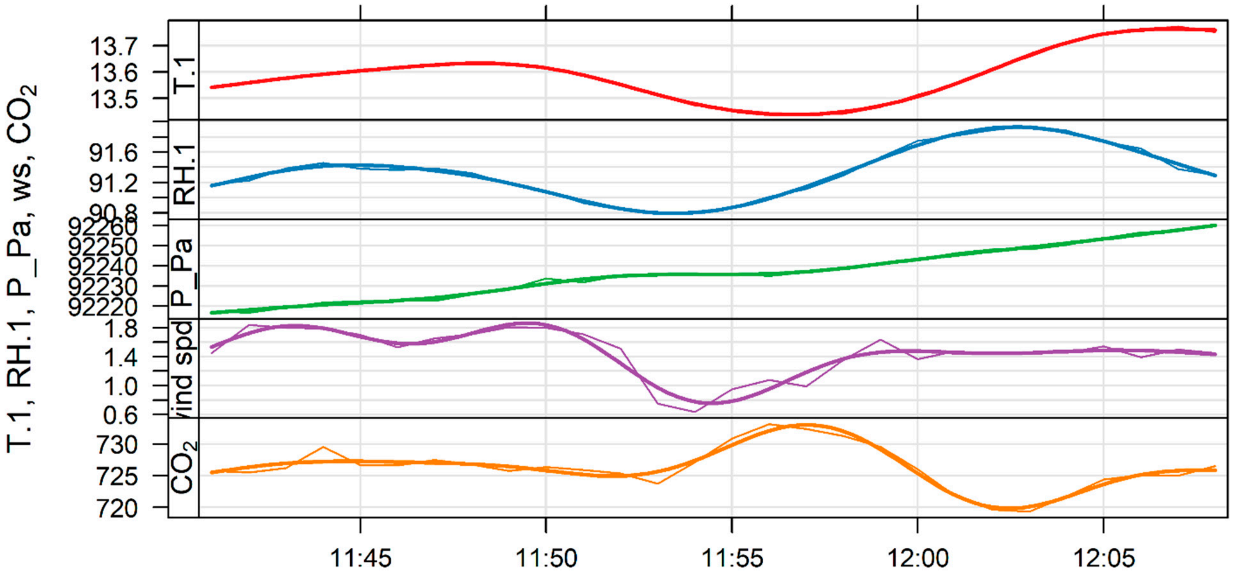

Figure S9. Detected environmental conditions (temperature in Celsius degrees, relative humidity in %, wind speed in m s^{-1}, and pressure in Pascal), and CO_2 concentrations in ppm at the downwind position on the ground in the first sampling day. The time format in the x-axis is CEST.

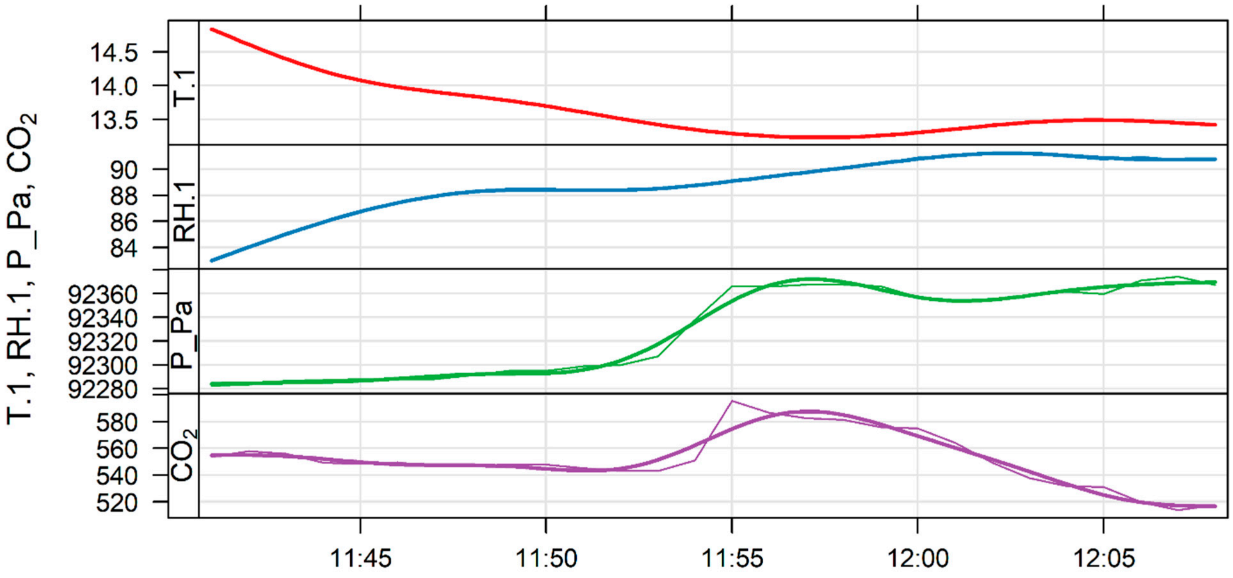

Figure S10. Detected environmental conditions (temperature in Celsius degrees, relative humidity in %, and pressure in Pascal) and CO_2 concentrations in ppm at the downwind position in flight (5.0 m ABGL) in the first sampling day. The time format in the x-axis is CEST.

Day 2

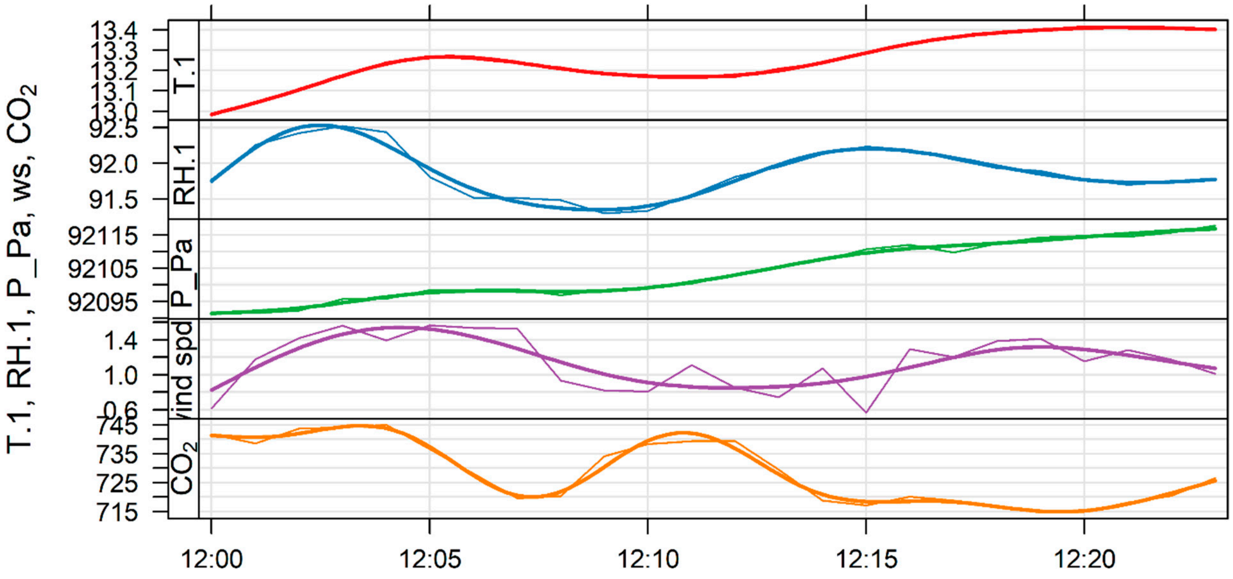

Figure S11. Detected environmental conditions (temperature in Celsius degrees, relative humidity in %, wind speed in m s^{-1}, and pressure in Pascal), and CO_2 concentrations in ppm at the downwind position on the ground in the second sampling day. The time format in the x-axis is CEST.

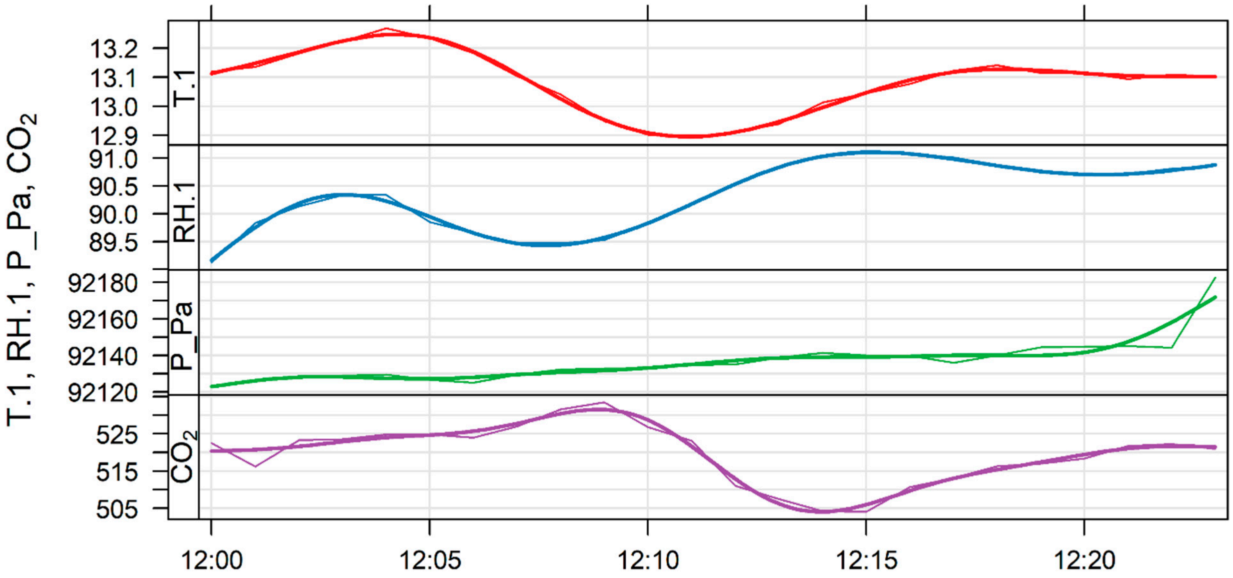

Figure S12. Detected environmental conditions (temperature in Celsius degrees, relative humidity in %, and pressure in Pascal) and CO_2 concentrations in ppm at the downwind position in flight (5.0 m ABGL) in the second sampling day. The time format in the x-axis is CEST.

Day 3

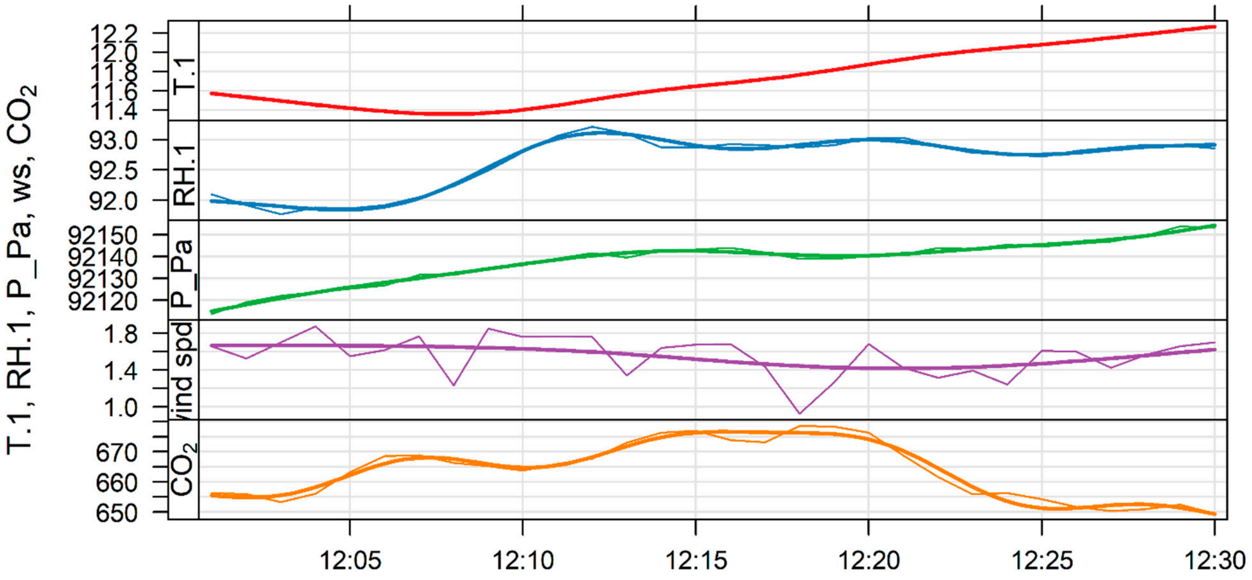

Figure S13. Detected environmental conditions (temperature in Celsius degrees, relative humidity in %, wind speed in m s^{-1}, and pressure in Pascal), and CO_2 concentrations in ppm at the downwind position on the ground in the third sampling day. The time format in the x-axis is CEST.

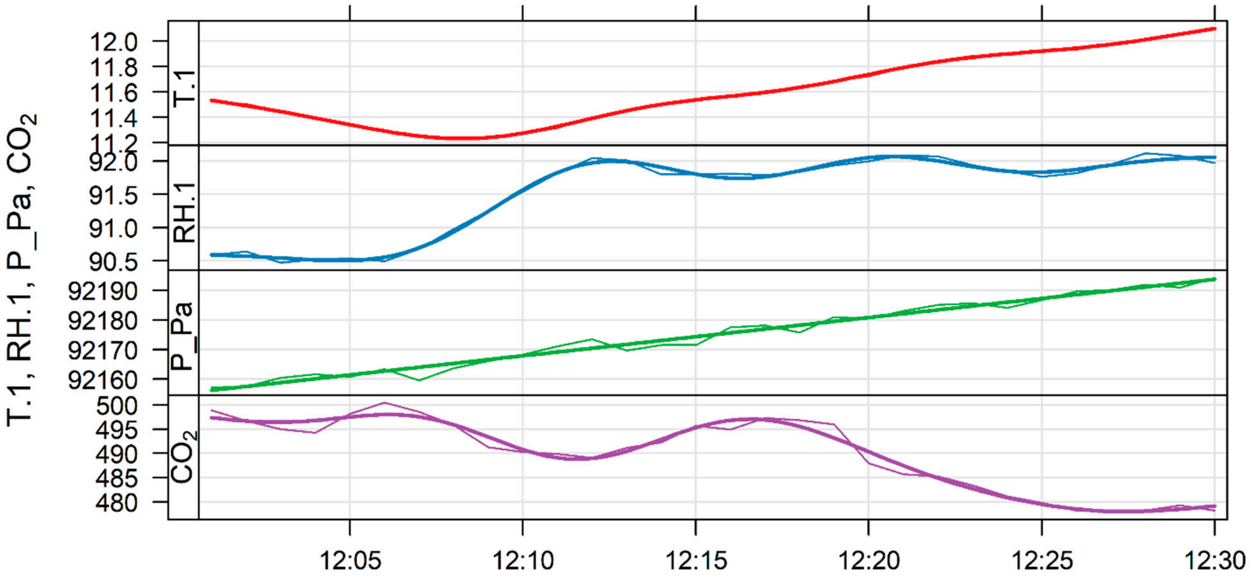

Figure S14. Detected environmental conditions (temperature in Celsius degrees, relative humidity in %, and pressure in Pascal) and CO_2 concentrations in ppm at the downwind position in flight (5.0 m ABGL) in the third day. The time format in the x-axis is CEST.

Day 4

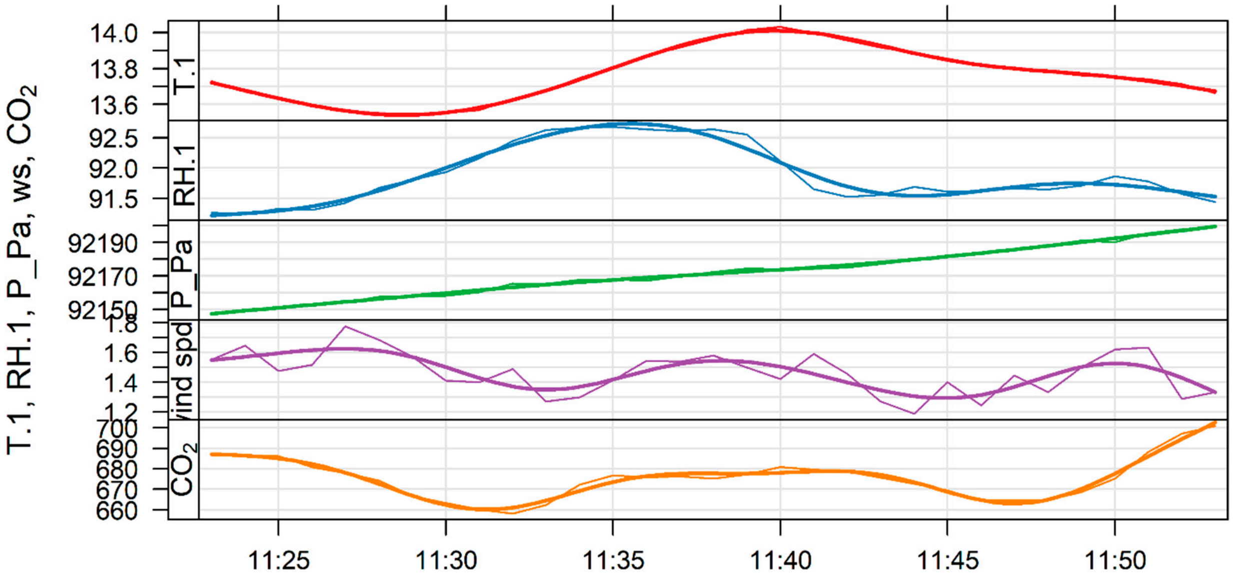

Figure S15. Detected environmental conditions (temperature in Celsius degrees, relative humidity in %, wind speed in m s^{-1}, and pressure in Pascal), and CO_2 concentrations in ppm at the downwind position on the ground in the fourth sampling day. The time format in the x-axis is CEST.

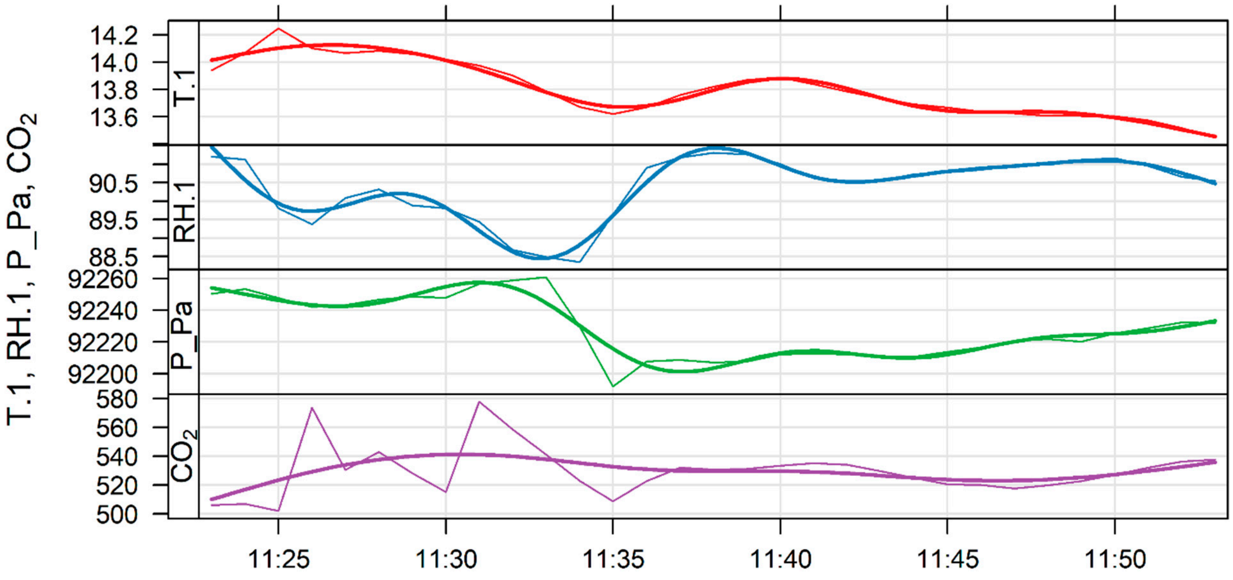

Figure S16. Detected environmental conditions (temperature in Celsius degrees, relative humidity in %, and pressure in Pascal) and CO_2 concentrations in ppm at the downwind position in flight (5.0 m ABGL) in the fourth day. The time format in the x-axis is CEST.

Day 5

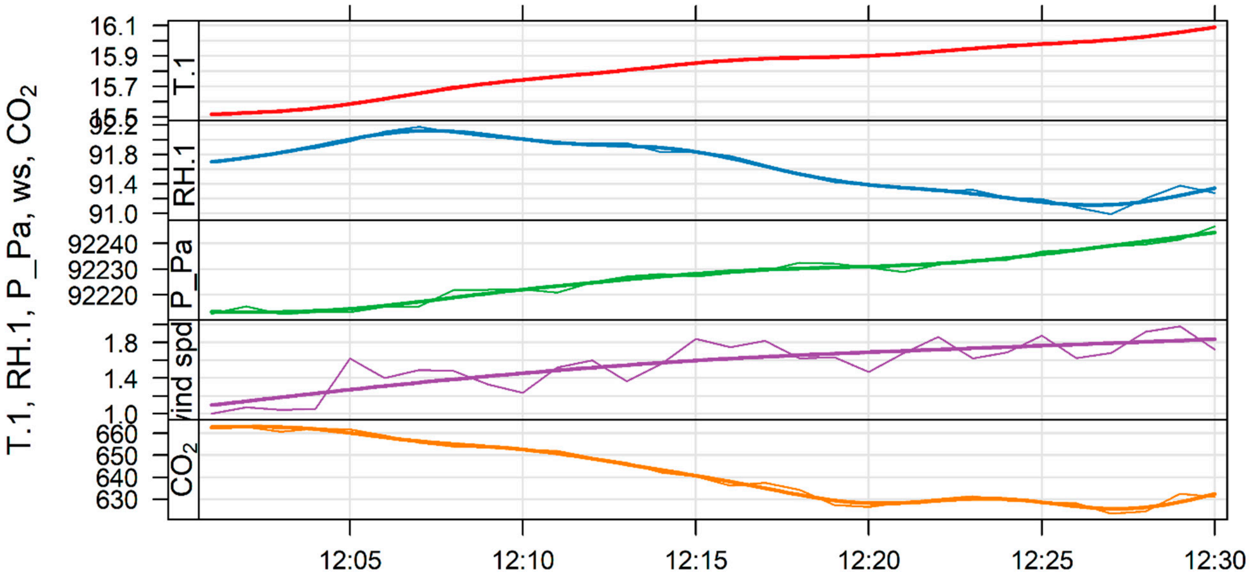

Figure S17. Detected environmental conditions (temperature in Celsius degrees, relative humidity in %, wind speed in m s^{-1}, and pressure in Pascal), and CO_2 concentrations in ppm at the downwind position on the ground in the fifth sampling day. The time format in the x-axis is CEST.

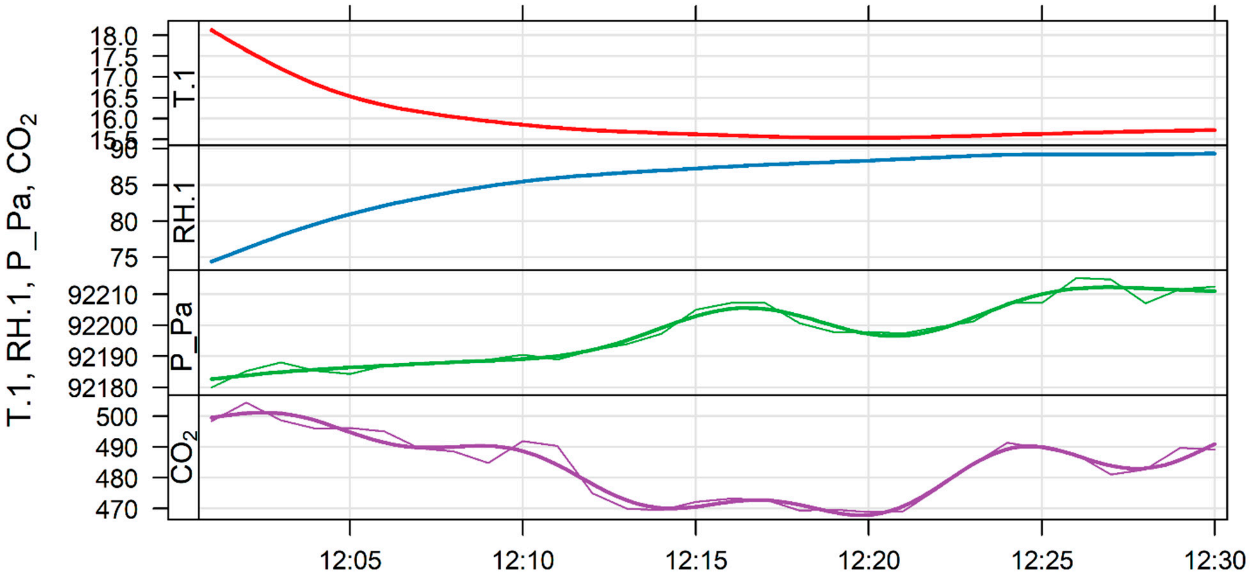

Figure S18. Detected environmental conditions (temperature in Celsius degrees, relative humidity in %, and pressure in Pascal) and CO_2 concentrations in ppm at the downwind position in flight (10.0 m ABGL) in the fifth day. The time format in the x-axis is CEST.

Day 6

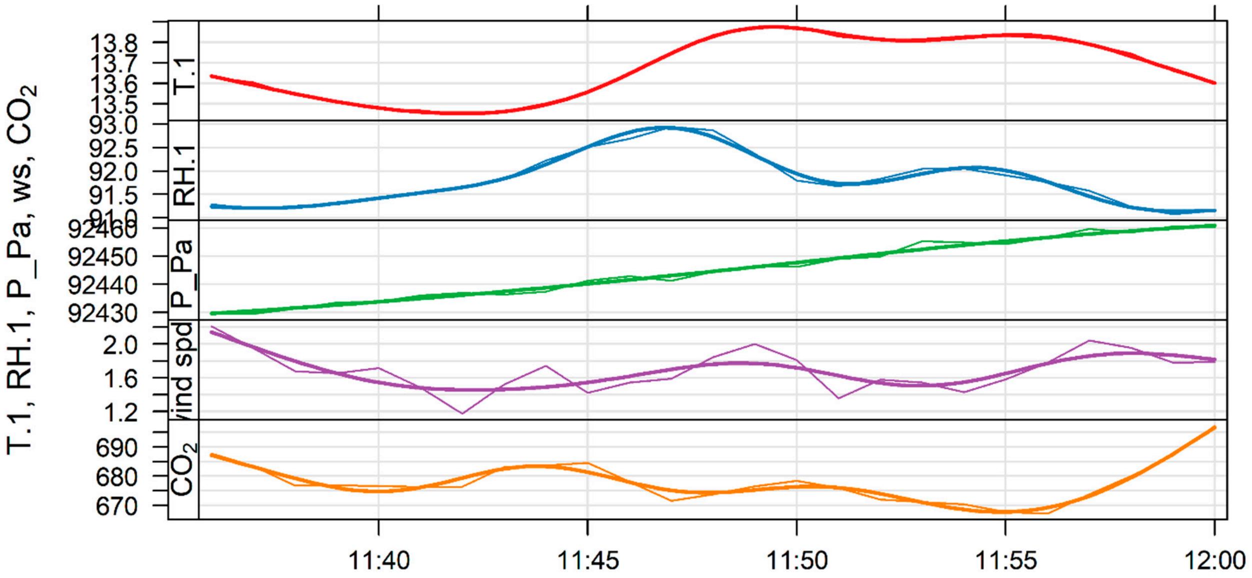

Figure S19. Detected environmental conditions (temperature in Celsius degrees, relative humidity in %, wind speed in m s^{-1}, and pressure in Pascal), and CO_2 concentrations in ppm at the downwind position on the ground in the sixth sampling day. The time format in the x-axis is CEST.

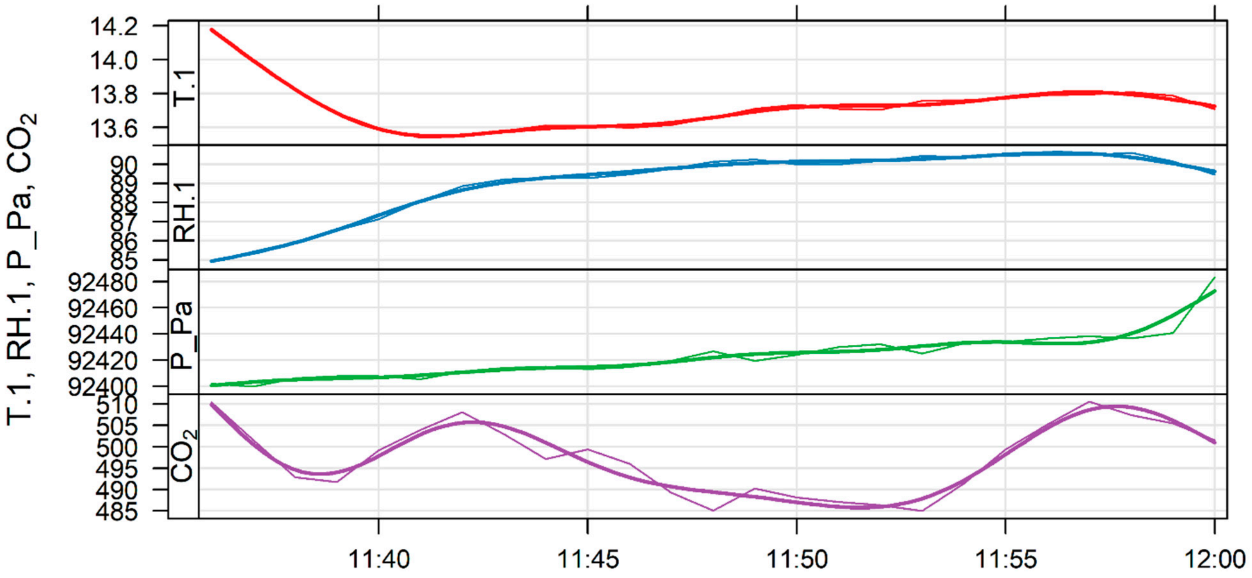

Figure S20. Detected environmental conditions (temperature in Celsius degrees, relative humidity in %, and pressure in Pascal) and CO_2 concentrations in ppm at the downwind position in flight (10.0 m ABGL) in the sixth day. The time format in the x-axis is CEST.

Day 7

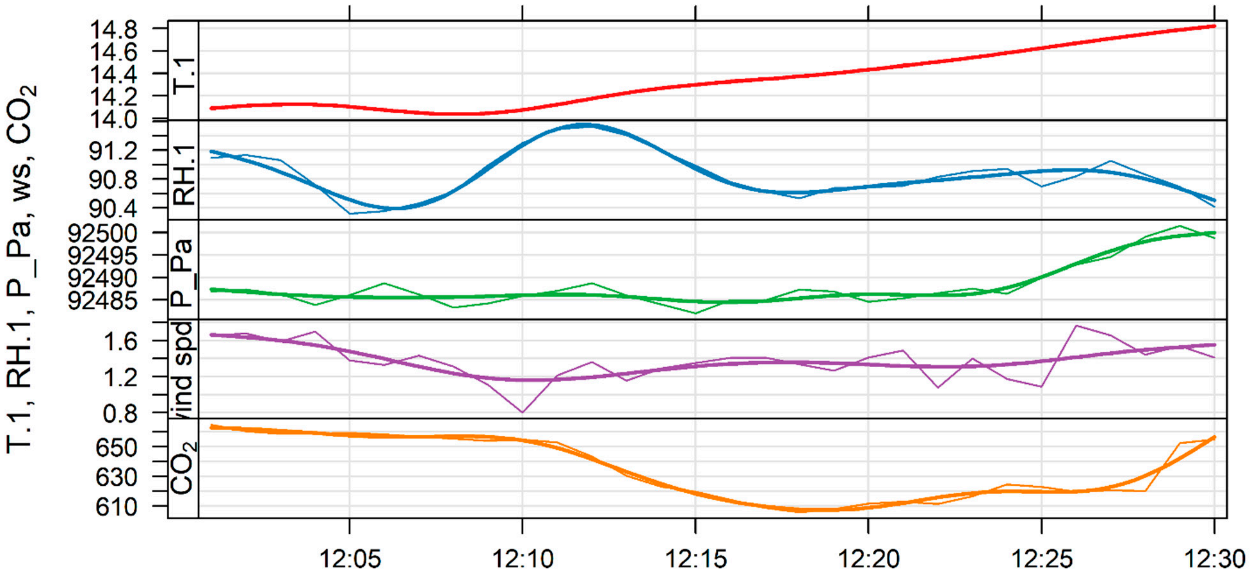

Figure S21 - Detected environmental conditions (temperature in Celsius degrees, relative humidity in %, wind speed in m s^{-1}, and pressure in Pascal), and CO_2 concentrations in ppm at the downwind position on the ground in the seventh sampling day. The time format in the x-axis is CEST.

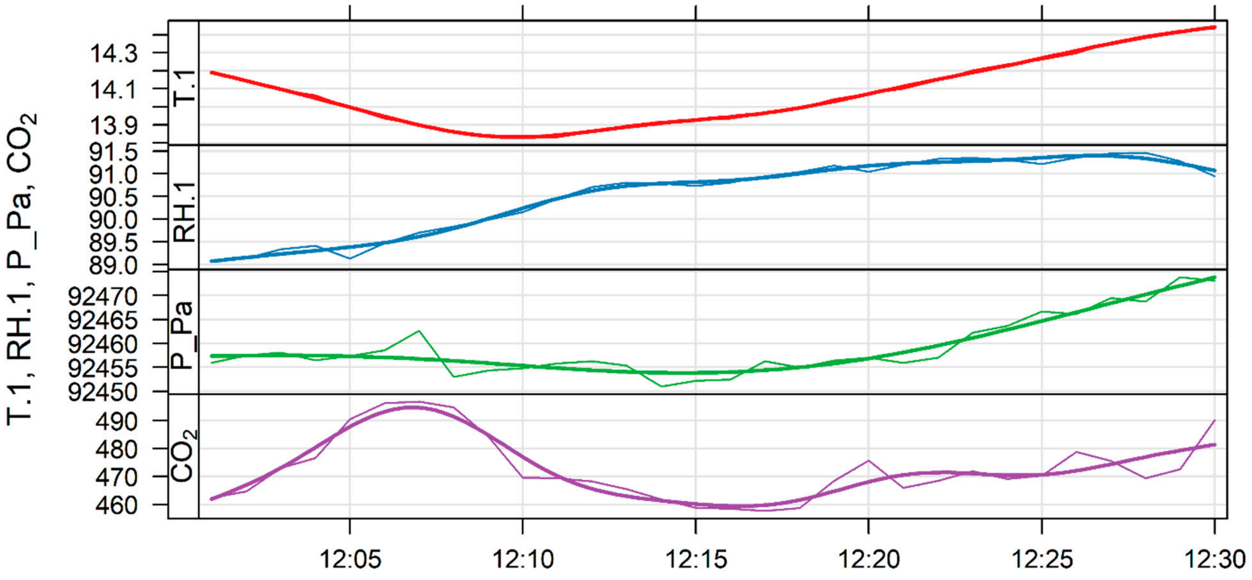

Figure S22 - Detected environmental conditions (temperature in Celsius degrees, relative humidity in %, and pressure in Pascal) and CO_2 concentrations in ppm at the downwind position in flight (10.0 m ABGL) in the seventh day. The time format in the x-axis is CEST.

Day 8

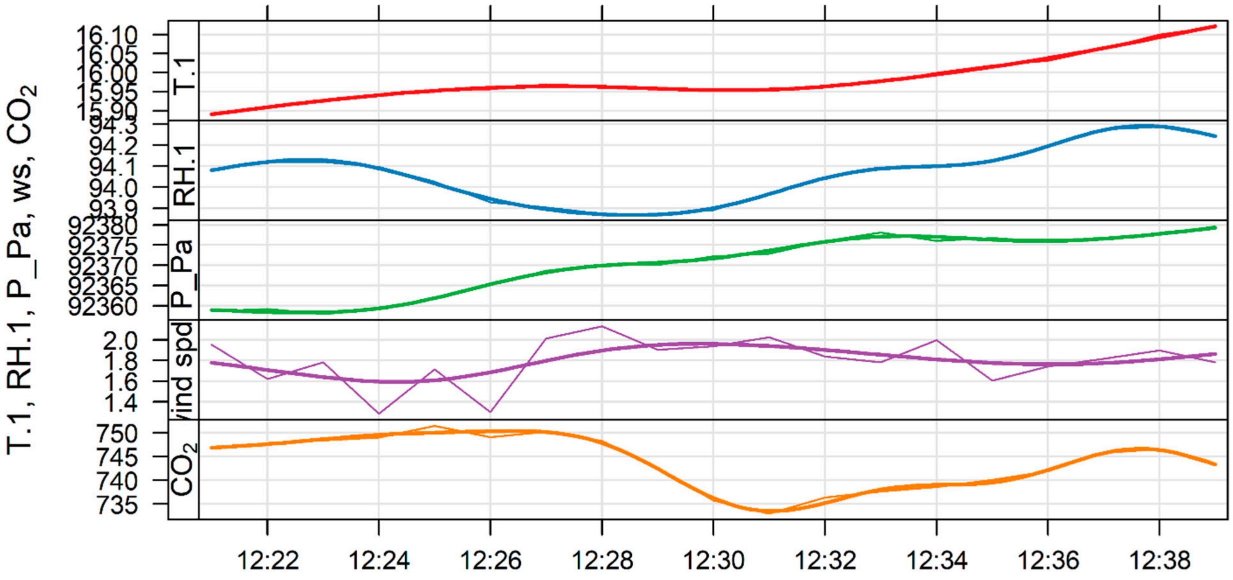

Figure S23 - Detected environmental conditions (temperature in Celsius degrees, relative humidity in %, wind speed in m s^{-1}, and pressure in Pascal), and CO_2 concentrations in ppm at the downwind position on the ground in the eighth sampling day. The time format in the x-axis is CEST.

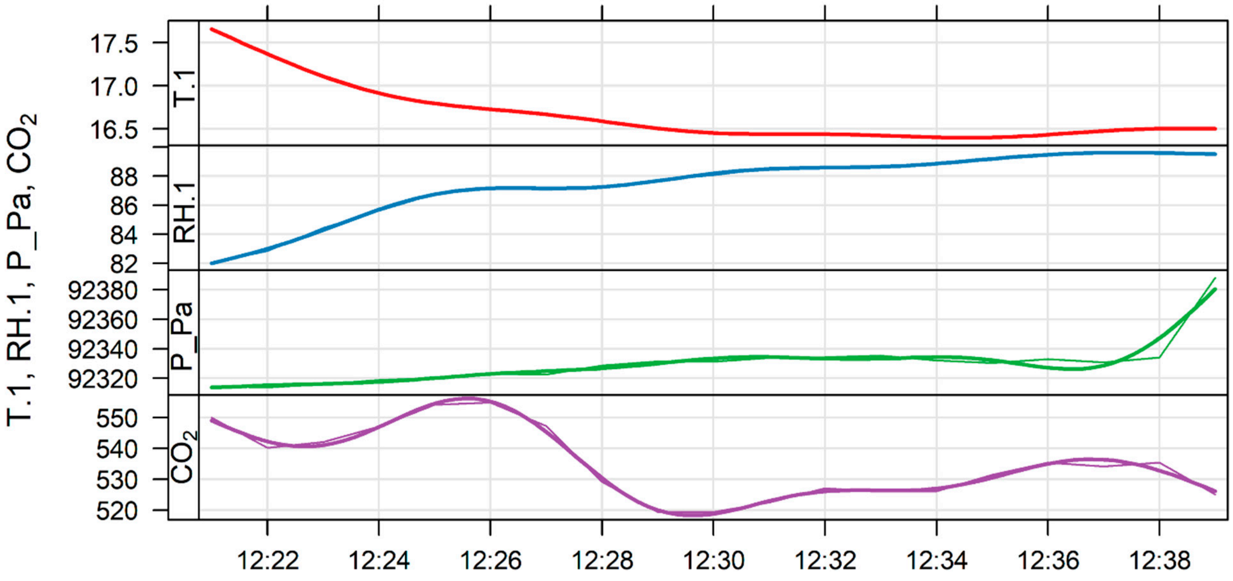

Figure S24 - Detected environmental conditions (temperature in Celsius degrees, relative humidity in %, and pressure in Pascal) and CO_2 concentrations in ppm at the downwind position in flight (10.0 m ABGL) in the eighth day. The time format in the x-axis is CEST.

Calculated CO_2 concentrations for each sampling day

Table S2. The five-number summary (minimum, first quartile, median, third quartile, and maximum values), including the mean value of the fifty calculated CO_2 concentrations (ppm) for each sampling day.

**Statistics**	**Day**							
	**1**	**2**	**3**	**4**	**5**	**6**	**7**	**8**
Minimum	587.00	593.90	592.10	556.80	549.00	598.50	554.80	610.50
1st quartile	587.00	593.90	592.10	556.80	549.00	598.50	554.80	610.50
Median	587.00	593.90	592.10	557.00	549.00	598.50	554.80	610.50
Mean	590.60	603.50	597.40	559.90	549.50	598.60	555.00	610.80
3rd quartile	590.10	602.00	595.60	559.70	549.10	598.50	554.90	610.50
Maximum	614.70	666.40	635.20	579.90	556.90	600.60	557.80	615.70
